# A Relay Network of Extracellular Heme-Binding Proteins Drives *C. albicans* Iron Acquisition from Hemoglobin

**DOI:** 10.1371/journal.ppat.1004407

**Published:** 2014-10-02

**Authors:** Galit Kuznets, Elena Vigonsky, Ziva Weissman, Daniela Lalli, Tsvia Gildor, Sarah J. Kauffman, Paola Turano, Jeffrey Becker, Oded Lewinson, Daniel Kornitzer

**Affiliations:** 1 B. Rappaport Faculty of Medicine, Technion – I.I.T. and the Rappaport Institute for Research in the Medical Sciences, Haifa, Israel; 2 CERM and Department of Chemistry, University of Florence, Sesto Fiorentino, Italy; 3 Microbiology Department, University of Tennessee, Knoxville, Tennessee, United States of America; University of Rochester, United States of America

## Abstract

Iron scavenging constitutes a crucial challenge for survival of pathogenic microorganisms in the iron-poor host environment. *Candida albicans*, like many microbial pathogens, is able to utilize iron from hemoglobin, the largest iron pool in the host's body. Rbt5 is an extracellular glycosylphosphatidylinositol (GPI)-anchored heme-binding protein of the CFEM family that facilitates heme-iron uptake by an unknown mechanism. Here, we characterize an additional *C. albicans* CFEM protein gene, *PGA7*, deletion of which elicits a more severe heme-iron utilization phenotype than deletion of *RBT5*. The virulence of the *pga7^−/−^* mutant is reduced in a mouse model of systemic infection, consistent with a requirement for heme-iron utilization for *C. albicans* pathogenicity. The Pga7 and Rbt5 proteins exhibit distinct cell wall attachment, and discrete localization within the cell envelope, with Rbt5 being more exposed than Pga7. Both proteins are shown here to efficiently extract heme from hemoglobin. Surprisingly, while Pga7 has a higher affinity for heme *in vitro*, we find that heme transfer can occur bi-directionally between Pga7 and Rbt5, supporting a model in which they cooperate in a heme-acquisition relay. Together, our data delineate the roles of Pga7 and Rbt5 in a cell surface protein network that transfers heme from extracellular hemoglobin to the endocytic pathway, and provide a paradigm for how receptors embedded in the cell wall matrix can mediate nutrient uptake across the fungal cell envelope.

## Author Summary

*Candida albicans*, a commensal fungus of human mucosal surfaces in healthy individuals, is a common cause of superficial infections, as well as of life-threatening systemic infections in individuals suffering from a reduced immune function. As a systemic pathogen, it has to cope with a scarcity of specific nutrients in the host environment, chief among them iron. To overcome this iron limitation, *C. albicans* is able to extract iron from heme and hemoglobin, the largest iron pools in the human body, via a pathway that involves endocytosis into the cell. Here we show that efficient heme uptake relies on a family of extracellularly-anchored proteins that serve as heme receptors, two of which, at least, are required for efficient heme utilization. Our data suggest the existence of a relay system that transfers heme from one protein to the next across the cell envelope, explaining the requirement for multiple heme receptors for efficient heme-iron utilization. This study extends our understanding of the pathway of host heme utilization by fungal pathogens, and provides new insights into the question of how nutrients such as heme cross the fungal cell wall.

## Introduction

*Candida albicans*, a commensal fungus normally residing on the skin and on mucosal surfaces, is commonly able to cause local mucosal, cutaneous and nail infections. In debilitated and immunocompromised patients, *C. albicans* is furthermore able to spread systemically and to cause deep-seated infections that can be life-threathening [1]. In order to survive and proliferate in host tissues, *C. albicans*, like all pathogenic microbes, needs to be able to acquire essential nutrients such as iron. Iron withholding has long been recognized as a defense mechanism deployed by the human host against invading microorganisms [2]. As a consequence, successful pathogens had to evolve mechanisms for scavenging iron from host molecules [3].

Several distinct iron acquisition mechanisms have been identified in *C. albicans* (reviewed in [4], [5]). High-affinity elemental iron uptake requires the permease Ftr1 [6], which is associated with a multicopper ferroxidase [7] that in turn relies on a copper pump, Ccc2, for its biogenesis in the Golgi [8], [9]. A siderophore-mediated iron uptake mechanism requires the hydroxamate type siderophore transporter Sit1/Arn1 [10], [11]. Ferritin-iron utilization depends on the adhesin Als3 [12]. Lastly, heme-iron utilization potentially enables *C. albicans* to gain access to the largest iron pool in the human body - some 70% of iron in the human body is heme iron, predominantly found in the oxygen carrier hemoglobin [13]. The heme iron acquisition pathway relies on Rbt5, an extracellular GPI-anchored heme receptor [14]. Rbt5 contains the fungal-specific CFEM domain, which is characterized by eight cysteine residues with conserved spacing [15]. Additional genes required for heme-iron utilization include Ca*HMX1*, encoding a heme oxygenase [16], [17], genes encoding components of the ESCRT system, and a Type I myosin involved in endocytic vesicle abscission, mutants of which are defective in both heme-iron endocytosis and heme-iron utilization [18]. A mutant of the vacuolar ATPase is defective only in heme-iron utilization, but not in endocytosis [18]. Together, these genetic studies suggested a pathway of heme-iron utilization in which interaction of free heme or hemoglobin with an extracellular receptor such as Rbt5 eventually leads to endocytosis, degradation of the protoporphyrin rings by heme oxygenase, and uptake of the released iron into the cytoplasm by a vacuolar iron permease.

Given the hemolytic capacity of *C. albicans* [19], hemoglobin utilization thus gives the organism access to an abundant iron reservoir during systemic infection. Nonetheless, *RBT5* was not required for virulence in a mouse model of systemic infection [20]. We noted however that *rbt5* ^−/−^ mutants are only partially defective in heme and hemoglobin utilization [14], suggesting that additional factors may exist alongside Rbt5 that mediate heme-iron transport across the cell envelope. Notably, missing from the model described above is a mechanism for transfer of heme or hemoglobin across the cell wall. The cell wall architecture is complex and not well understood, but is thought to consist of an inner core of β-(1,3)- and β-(1,6)-linked glucans and of chitin (β-(1,4)-N-acetylglucosamine), and an outer layer of glycoproteins [21]. This provides a porous structure, which allows for diffusion of solutes and macromolecules, though the exact size limit for diffusion of solutes, and whether facilitated diffusion can occur, is not known [22]. Although a significant fraction of Rbt5 can be found in the membrane, the majority of Rbt5 is covalently linked to the cell wall, where it represents one of the most prominent proteins, particularly under iron starvation conditions ([23], [24] and this work). How such a cell wall matrix-embedded heme-binding protein could facilitate transport of hemin or hemoglobin across the cell wall was unclear.

Here, we characterize a new heme-binding CFEM protein, Pga7, that, although less abundant, is functionally more important than Rbt5 for heme-iron utilization in culture, and that contributes to virulence in a mouse model of infection. *In vivo*, Pga7 is localized to the cell envelope, at an internal position compared to Rbt5. *In vitro*, both Rbt5 and Pga7 are able to extract heme from hemoglobin, and furthermore, heme can be rapidly transferred between the two CFEM proteins, suggesting that they form the basis of a heme relay system across the cell envelope.

## Results

### Pga7 is essential for heme-iron utilization

Of the two extracellular *C. albicans* proteins, Rbt5 and Rbt51, that were previously shown to bind hemin, only the *rbt5* ^−/−^ mutant was defective in heme-iron utilization, and this defect was only partial, since an increase in hemoglobin concentration could restore growth [14]. However, genomic analysis revealed that besides Rbt5 and Rbt51, *C. albicans* contains several additional related CFEM proteins, including Csa1/Wap1, Pga7, and Csa2 (Fig. S1), as well as more distantly related CFEM proteins such as Ssr1. Csa1 had been previously analyzed and was not found to affect hemoglobin utilization under our standard growth conditions [14], [20]. We next focused on Pga7 because we found that like *RBT5*, *PGA7* is transcriptionally induced under iron starvation conditions (our unpublished results and [25]). We deleted both alleles of *PGA7* in a wild-type background as well as in a strain lacking *RBT5*, to identify possible epistatic relationships among these genes.

**Figure 1:**
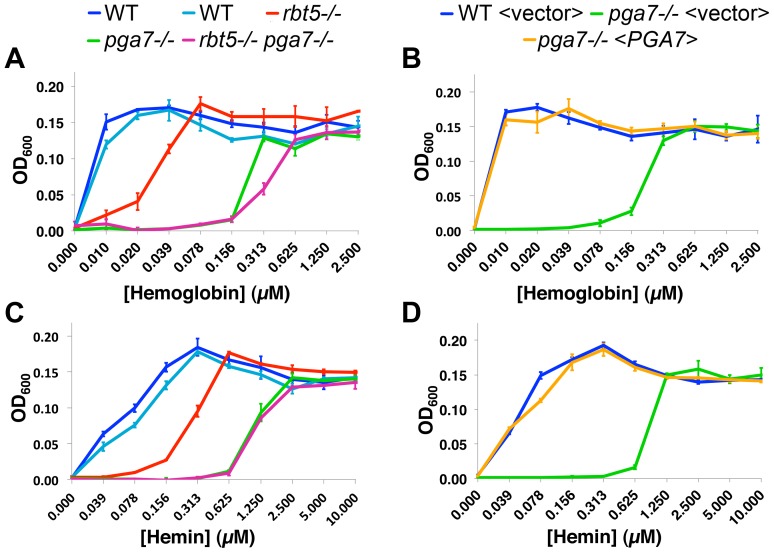
Pga7 plays a central role in heme and hemoglobin-iron utilization in *C. albicans*. *PGA7* and *RBT5* deletion strains (A, C) or *PGA7* re-integrant strains (B, D) were grown in iron-free RPMI medium in the presence of increasing concentration of either human hemoglobin (A, B) or hemin (C, D) as a sole source of iron. Optical density was measured after 3 days at 30°C. Error bars represent standard deviations of triplicates. The following strains were used: A, C: CAI4 (KC79, KC590)), KC589 (*rbt5* ^−/−^), KC626 (*pga7^−/−^*), KC594 (*rbt5* ^−/−^ *pga7^−/−^*). The two wild-type starting strains from different sources were highly concordant. B, D: KC645 (WT<vector>), KC646 (*pga7^−/−^*<vector>), KC647 (*pga7^−/−^*<*PGA7*>).

As shown in Fig. 1A, the *pga7* ^−/−^ mutant exhibited a defect in hemoglobin utilization that was more profound than the defect exhibited by the *rbt5* ^−/−^ strain: the *pga7* ^−/−^ *RBT5^+/+^* strain requires some 30-fold higher hemoglobin concentration than the wild-type strain to reach the same level of growth, vs. 10-fold higher requirement for the *rbt5* ^−/−^ *PGA7^+/+^* mutant. Reintroduction of *PGA7* under its native promoter into the *pga7^−/−^* mutant was sufficient to restore normal growth with hemoglobin as sole source of iron (Fig. 1B), confirming that deletion of this gene was responsible for the hemoglobin utilization defect of the *pga7^−/−^* strain. The double *pga7* ^−/−^ *rbt5* ^−/−^ mutant appears only slightly more defective in hemoglobin utilization than the *pga7* ^−/−^ mutant by itself (Fig. 1A), arguing for a central role for Pga7 in the pathway. The same order of growth of the strains was observed when hemin was used as sole iron source (Fig. 1C, 1D), with similar ratios between the minimal concentrations of hemin required for growth of the different mutants. Notably, approximately four-fold higher hemin concentrations were required to reach the same growth levels compared to hemoglobin, consistent with the 4 heme-per-hemoglobin stoichiometry.

The relative effects of deleting *PGA7* and *RBT5* on heme-iron utilization were reproduced in the *ccc2^−/−^* background (Fig. S2), which is defective in high-affinity iron uptake [8]. This attests to the robustness of the effects of these mutants on heme-iron utilization, and confirms that the pathway in which these proteins participate is distinct from the high-affinity iron uptake pathway of *Candida*. Deletion of the CFEM protein-encoding genes *CSA1* and *RBT51*, which did not affect heme utilization in the presence of *PGA7* [14], still did not affect heme utilization even in the absence of *PGA7* (Fig. S2), indicating that they are not responsible for the residual heme utilization of the *pga7^−/−^* mutant.

Interestingly, a small but reproducible difference between the *pga7* ^−/−^ *RBT5^+/+^* strain and the double *pga7* ^−/−^ *rbt5* ^−/−^ mutant could be detected with hemoglobin (Fig. 1A and S2A), but not with hemin as sole iron source (Fig. 1C and S2B), raising the possibility of a specific role for Rbt5 in heme-iron acquisition from hemoglobin.

### *PGA7* contributes to virulence in a mouse model of systemic candidiasis

**Figure 2:**
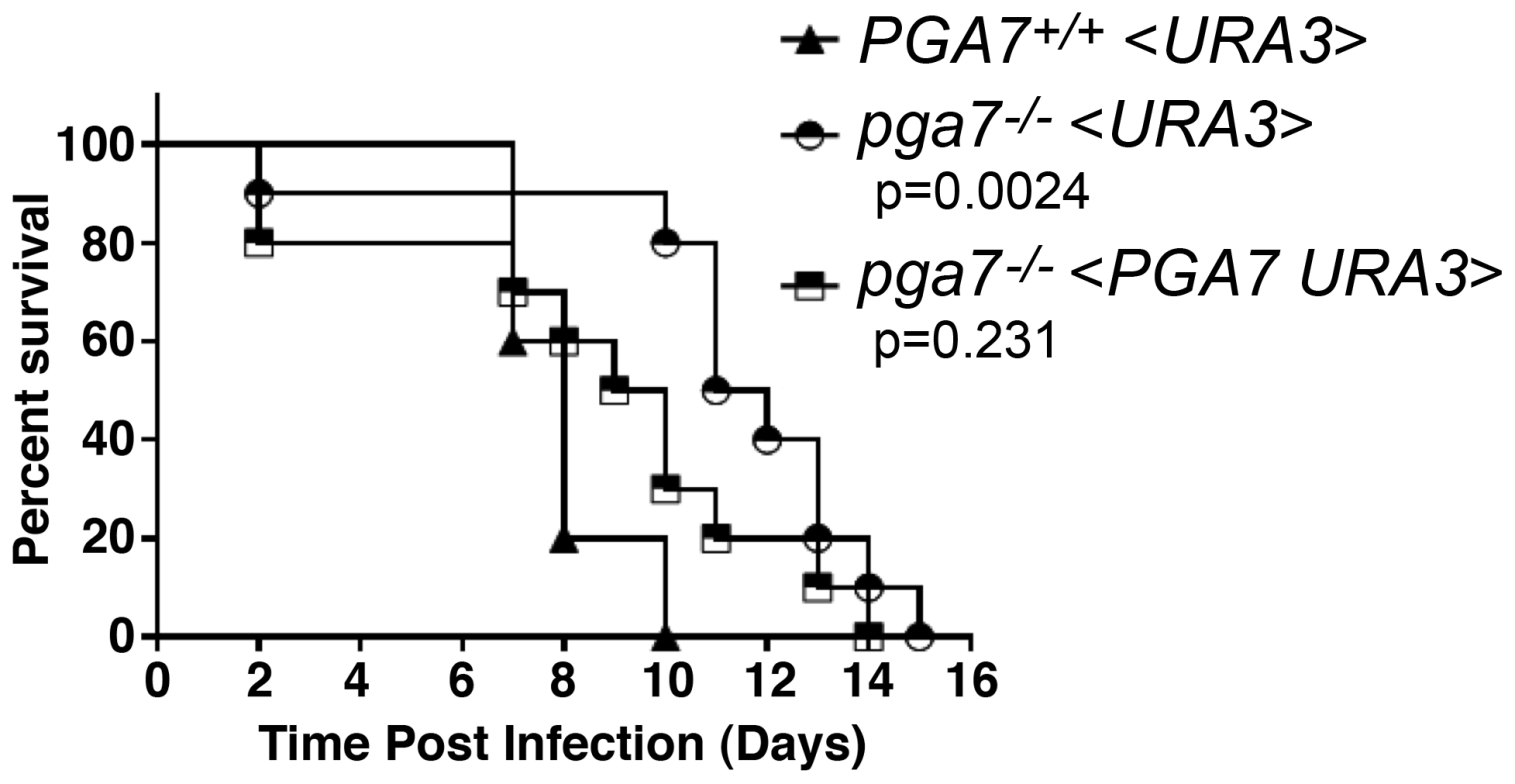
The *pga7^−/−^* strain is less virulent in a mouse model of systemic candidiasis. Survival of intravenously challenged mice with *Candida albicans* strains is indicated. Groups of 5 mice were inoculated with 2 independent clones of *pga7^−/−^* (KC646) or re-integrant (KC647) and were grouped together and compared to the wild type (KC645). Mice infected with the *pga7^−/−^* strains survived significantly longer that mice infected with the wild type strain (p = 0.0024), whereas mice infected with the *PGA7* re-integrant strains did not survive significantly longer (p = 0.2310).

High-affinity elemental iron acquisition was reported to be important for *C. albicans* pathogenicity [6]. We took advantage of the strong heme-iron utilization defect of the *PGA7* deletion *in vitro*, to test whether *C. albicans* heme acquisition contributes to its pathogenicity as well. We used a mouse model of systemic infection, and compared the survival rate of mice infected with the WT strain, the *pga7^−/−^* strain, and *pga7^−/−^ <PGA7>* re-integrant strain. Mice infected with the parental *PGA7^+/+^* strain died significantly faster than the mice infected with the *pga7^−/−^* strains (p = 0.0024) (Fig. 2), but not significantly faster than the mice infected with the re-integrant strains (p = 0.23), indicating that Pga7 functions as a virulence factor in a mouse model of systemic candidiasis. This difference in survival rates suggests that even in the presence of a functional high-affinity iron uptake system, heme-iron acquisition also contributes to *C. albicans* virulence.

### Different abundance of Rbt5 and Pga7

**Figure 3:**
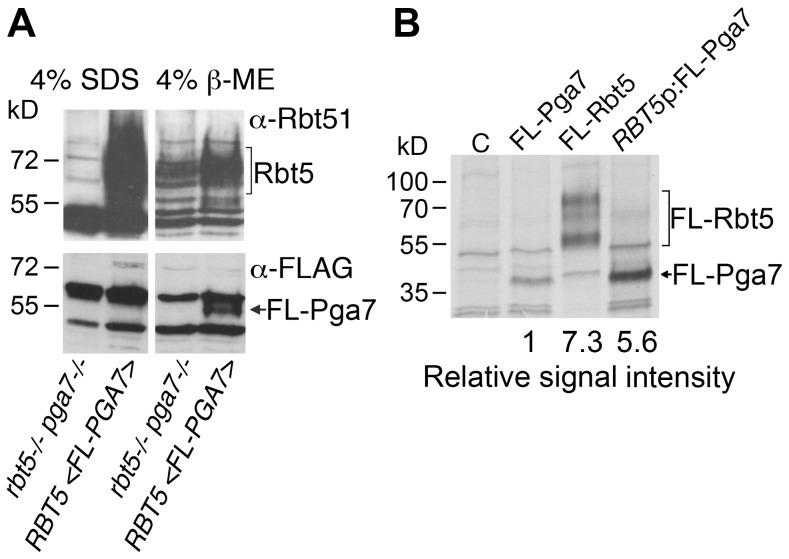
Rbt5 is more abundantly expressed than Pga7. (A) Western blot detection of cell envelope fractions of cells expressing FLAG-tagged Pga7 (KC605) or native Rbt5 under iron starvation conditions, vs. a control lacking both *RBT5* and *PGA7* (KC594). Left panels: SDS extract of the cell pellet after mechanical lysis. Right panels: 4% ß-ME extract of the cell pellet remaining after SDS extraction. Top panels: detection of the membrane with α-Rbt51, which cross-reacts with Rbt5 (Rbt51 itself is very weakly expressed under these conditions [14]). Bottom panels: detection of the membrane with monoclonal α-FLAG. (B) Expression of FLAG-tagged Rbt5 (KC706) or Pga7 (KC605), or FLAG-tagged Pga7 expressed under the RBT5 promoter, (KC712) in *C. albicans* cells grown under iron-limiting conditions (YPD +1 mM ferrozine), was measured by labeling the cells with ^35^S-methionine/^35^S-cysteine, followed by immunoprecipitation using a rabbit anti-FLAG antiserum, and separation of the proteins by SDS-PAGE. C = control cells with no FLAG.

The observation that, in spite of the high homology between the Pga7 and Rbt5 proteins (Fig. S1), the *pga7^−/−^* strain had a more severe heme-iron uptake phenotype than the *rbt5^−/−^* strain, prompted us to test whether the severity of the mutant phenotypes correlated with the relative levels of each protein in the cell. Pga7 was tagged with a FLAG tag introduced just beyond the predicted signal peptide cleavage site, and expressed in cells deleted for *PGA7* but expressing *RBT5*. Expression of *RBT5* and of *FLAG-PGA7* was induced by iron starvation, and the cells were mechanically lysed and pelleted. No FLAG-Pga7 could be detected in the cell supernatant (not shown). Extraction of the salt-washed cell pellet with hot SDS released a large amount of Rbt5, as detected using the cross-reacting α-Rbt51 antiserum [14], but no FLAG-reactive signal (indicative of FLAG-Pga7) was detected above background (Fig. 3A). Treatment of the remaining pellet with a reducing agent (β-mercaptoethanol; β-ME) released some additional Rbt5, but most predominantly, a clear band representing FLAG-Pga7 was now detected. This suggests that Pga7 is attached to the cell wall by β-ME -sensitive bonds.

The difficulty of detecting Pga7 suggested a low level of expression. In order to directly compare protein expression rates of Pga7 and Rbt5, we used a FLAG epitope-tagged version of each protein, thereby eliminating variations due to different antisera or different epitopes. The epitope sequence was inserted at a similar location in both proteins, right after the predicted signal peptide cleavage site, i.e. at the N-terminus of the mature proteins (Fig. S1). FLAG-tagged Pga7 was also cloned under the presumably stronger *RBT5* promoter. To ascertain that addition of the FLAG epitope, or expression of FLAG-Pga7 under the stronger *RBT5* promoter, did not interfere with the activity of the proteins, we tested complementation of the *rbt5^−/−^* and *pga7^−/−^* mutants with the tagged vs. non- tagged constructs. FLAG-Rbt5 was able to complement the heme-utilization defect of the *rbt5^−/−^* strain as efficiently as non-tagged Rbt5. Similarly, FLAG-Pga7, both under its native promoter and under the *RBT5* promoter, was able to fully complement the *pga7^−/−^* mutant for heme iron utilization, suggesting that the tagged proteins are fully active (Fig. S3).

Protein expression levels of these three constructs were compared in iron-starved cells by pulse-labeling for 5 min with ^35^S-methionine/^35^S-cysteine, followed by immunoprecipitation of the newly synthesized proteins with anti-FLAG antibodies. Quantitation of the FLAG-Rbt5 and FLAG-Pga7 signals revealed that Rbt5 is expressed at approximately an order of magnitude higher levels than Pga7 under its native promoter. When Pga7 was expressed under the *RBT5* promoter, it reached levels of expression similar to those of Rbt5 (Fig. 3B). Thus, the stronger phenotype of the deletion mutant of *PGA7* did not correlate with its expression levels.

### Rbt5 and Pga7 exhibit distinct localization and cell wall attachment

The requirement for these two similar proteins in the pathway of heme iron acquisition might be explained by a different subcellular localization. The experiment shown in Fig. 3A suggested that Rbt5 and Pga7 are differently distributed in the cell envelope. However, we found that the lower levels of expression of Pga7 made detection and meaningful comparison of its localization with that of Rbt5 difficult. Therefore, in the following experiment, we compared the subcellular localization of FLAG-Pga7 and FLAG-Rbt5 expressed at similar levels under the *RBT5* promoter (Fig. 3B), both by immunofluorescence microscopy, and by differential cell extraction.

**Figure 4:**
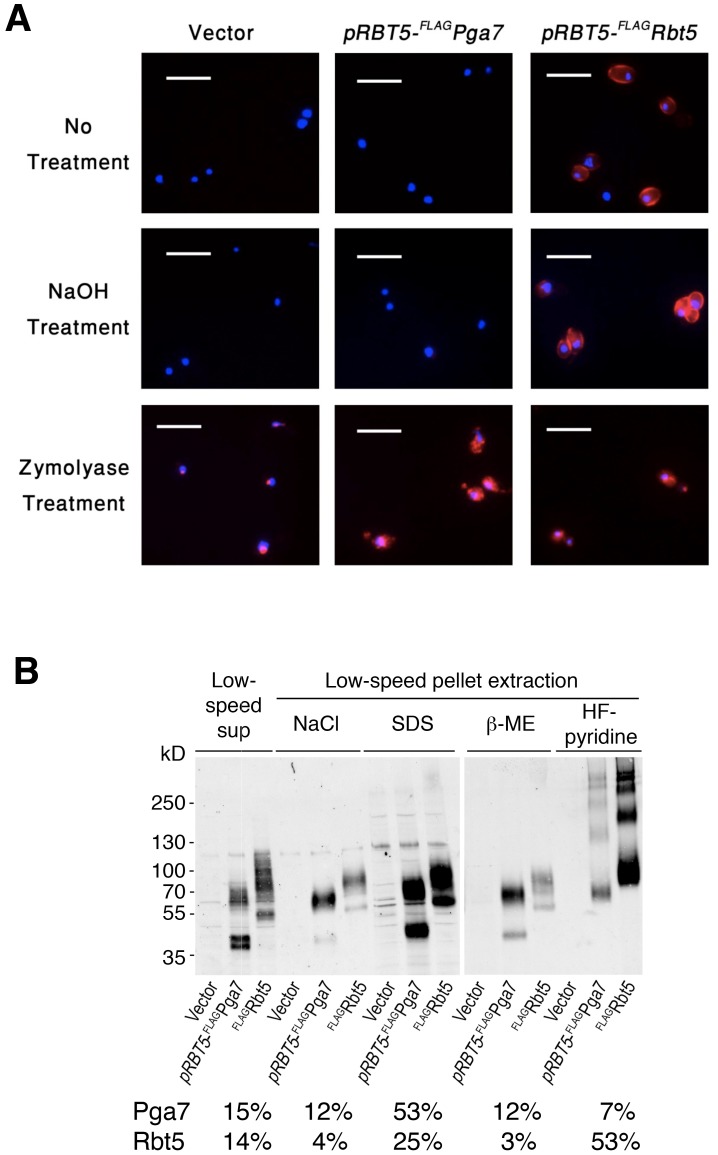
Different localization of Pga7 and Rbt5 expressed at similar levels. (A) Cells expressing either FLAG-tagged Rbt5 (KC713) or FLAG tagged Pga7 under the *RBT5* promoter (KC711), and a control wild type strain (KC68), were grown under iron limiting condition and fixed with formaldehyde. The fixed cells were either washed in PBS (“no treatment”), incubated with 250 mM NaOH (“NaOH treatment”), or fully permeabilized with zymolyase, β-ME and methanol (“Zymolyase treatment”). The cells were then incubated with anti-FLAG antibody followed by anti-Mouse IgG-Cy3 coupled antibody. red = FLAG, blue = DAPI. Bars = 10 µm. (B) To investigate the membrane or cell wall attachment mode of Rbt5 and Pga7, sequential fractionation and extraction of the cell culture shown in A was performed. An equal relative amount of each fraction was loaded in each lane, and the proteins were detected by immunoblotting with a rabbit anti-FLAG antibody. The FLAG-Rbt5 or FLAG-Pga7 signal was quantitated in each lane. The total amount of each protein was obtained by combining the signals of all the fractions together. The relative amount of protein in each given fraction, compared to the total for that protein, is indicated below the gel. The total signal of FLAG-Pga7 was about 15% less than the total signal of FLAG-Rbt5.

Immunofluorescence microscopy in cells permeabilized by partial cell wall digestion and membrane solubilization revealed that the FLAG epitope of both proteins was detectable throughout the cells in similar amounts, based on signal strength (Fig. 4A, “Zymolyase treatment”). Biochemical analysis of the same cultures used for immunofluorescence (see below, Fig. 4B) confirmed that the total cellular amounts of both proteins were similar. Nonetheless, while in untreated cells the anti-FLAG antibody clearly decorated the circumference of the cells expressing FLAG-Rbt5, no signal was detected in cells expressing FLAG-Pga7 (Fig. 4A, “No treatment”). Treatment of the cells with NaOH led to a stronger FLAG-Rbt5 signal, possibly by causing partial removal of the glycosyl moieties and alkali-sensitive ester-linked proteins at the cell wall perimeter [14], [23], allowing better access for the antibodies to the exposed epitopes. However, NaOH treatment still did not reveal a signal in the FLAG-Pga7-expressing cells (Fig. 4A, “NaOH treatment”). This experiment indicates that Rbt5 and Pga7 differ in their localization, with Rbt5 being exposed at the periphery, and Pga7 located more internally, either buried within the cell wall or bound to the cell membrane.

Pga7 and Rbt5 both display the modular organization and sequence elements characteristic of GPI-anchored proteins (Fig. S1), predictive of either membrane attachment or covalent linking to the cell wall [26]. In order to identify biochemical differences between Rbt5 and Pga7 cell envelope attachment that may underlie their distinct localizations, we used a series of sequential extractions to distinguish between soluble or membrane-bound vs. cell wall-bound pools of Pga7 and Rbt5, and to identify the mode of attachment to the cell wall. Different attachment modes of proteins to the cell wall can be disrupted by distinct treatments [27]. Non-covalent attachment via salt bonds is efficiently disrupted by NaCl, while GPI-anchored proteins covalently linked via a glycosidic bonds to the ß-(1,6)-linked sugars can be extracted with hydrogen fluoride-pyridine (HF-pyridine; [23]), and proteins covalently linked via disulfide bonds to the cell wall can be solubilized by treatment with β-ME. Thus, after mechanical lysis with glass beads of the cells expressing the FLAG-tagged proteins, the supernatants were separated by centrifugation, and the pellets were further extracted sequentially with 1 M NaCl, 2% SDS, and 4% β-ME. Finally, the remaining cell wall pellets were treated with HF-pyridine. Equal relative amounts of each fraction were analyzed side-by-side by polyacrylamide gel electrophoresis followed by Western blotting with anti-FLAG.

While both Rbt5 and Pga7 could be detected in all the fractions, the respective proportions of each protein were different (Fig. 4B). Rbt5 was best visible in the HF-pyridine extractable fraction, followed by the SDS-extractable fraction. In contrast, Pga7 was most abundant in the SDS-extractable fraction, followed by the supernatant and salt- and β-ME -extractable fractions. These results suggest that Rbt5 is mainly distributed between a membrane-embedded (SDS-extractable) and a sugar-linked cell wall-attached (HF-sensitive) pool. Pga7, in contrast, is mainly found in a cell membrane (SDS-extractable) - embedded pool, but also exhibits a sizeable disulfide cross-linked (β-ME-sensitive) cell wall pool. The different cell envelope attachment distribution of these two proteins could thus account for the different subcellular localization, as detected by immunofluorescence microscopy of the cells (Fig. 4A).

It should be noted that the cellular distribution of Pga7 detected upon overexpression under the *RBT5* promoter may not reflect the distribution of the natively expressed protein. Indeed, while the levels of natively expressed FLAG-Pga7 are much lower, the only fraction where the protein is consistently detected is the β-ME-sensitive cell wall fraction (Fig. 3A). This suggests that the accumulation of Pga7 in the SDS-extractable fraction seen in Fig. 4B may be an artifact of overexpression, possibly due to saturation of the cell wall protein partners that bind Pga7 via disulfide bonds. Nonetheless, both natively expressed and overexpressed Pga7 differ in their cell envelope attachment from natively expressed Rbt5. This different cell envelope attachment could account for their differential localization (Fig. 4A), with Rbt5 being primarily a GPI-anchored outer cell wall protein and Pga7 primarily a disulfide bond-linked inner cell wall protein, and consequently for their distinct functions (Fig. 1).

### Pga7 exhibits a higher affinity for heme than Rbt5

Rbt5 was previously shown to bind heme [14]. To test whether Pga7 similarly binds heme, and to compare its heme binding to that of Rbt5, both proteins were produced heterologously in the fungal *Pichia pastoris* secretion system, which is expected to closely mimic the biogenesis pathway of these proteins in *C. albicans*. The expressed protein fragments, Pga7_18–195_ and Rbt5_23–219_, extend from the predicted signal peptide cleavage site to the predicted GPI anchor addition site of each protein.

**Figure 5:**
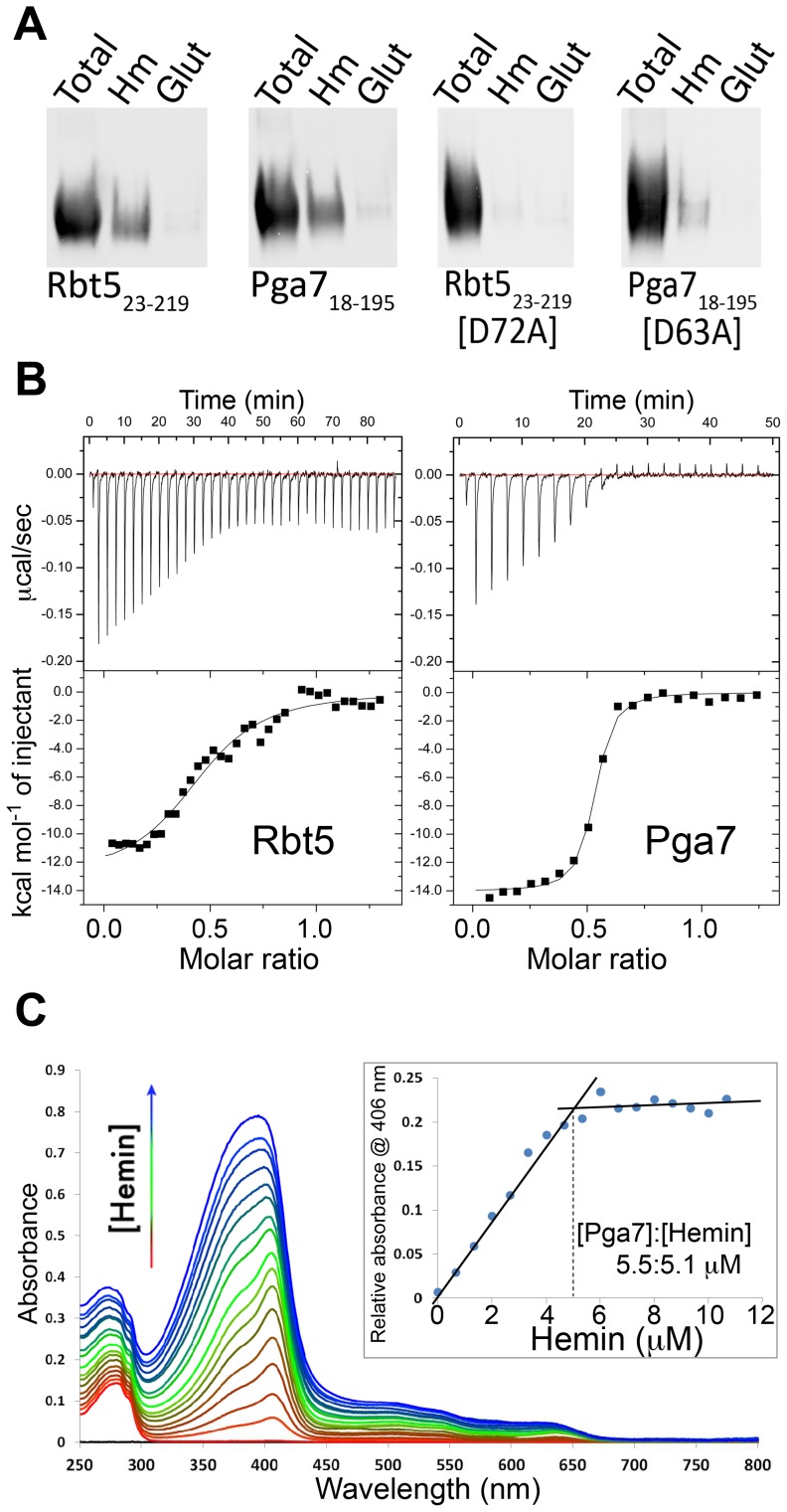
Interaction of Pga7 and Rbt5 with heme. (A) Recombinant wild type Rbt5_23–219_ and Pga7_18–195_ or their mutants in a conserved aspartic acid residue were incubated with hemin agarose (Hm) or glutathione agarose (glut) beads. The beads were washed and bound proteins were released by heating to 100°C in SDS gel loading buffer, separated by PAGE and detected by Western blotting with an anti-Myc antibody. (B) ITC analysis of the interaction of hemin with Rbt5_23–219_ (left panel), and with Pga7_18–195_ (right panel). The proteins (60 µM) were titrated into 10 µM hemin (Pga7) or into 20 µM hemin (Rbt5). Representative experiments are shown, with the heat signal in the top half of each panel and the binding isotherm derived from this signal in the lower half. The average fitting of three independent experiments with Rbt5-heme gave *n* = 2.152±0.0706 sites, *Ka* = 1.852×10^5^±3.679×10^4^ M^−1^ and *H* = −7454±304.7 KJ mol^−1^. The average fitting of three independent experiments with Pga7-heme gave *n* = 1.930±0.00999 sites, *Ka* = 1.531×10^7^±1.817×10^6^ M^−1^ and *H* = −7002±56.69 KJ mol^−1^. (C) UV/visible spectra from the titration of 1 µl hemin aliquots (2 mM hemin stock) into 3 ml of 5.5 µM apoPga7_18–195_ (1 cm path length cuvette, PBS). The inset shows the hemin-Pga7 absorbance minus hemin alone absorbance at 406 nm, at increasing hemin concentrations. The inflection point indicates saturation of hemin binding to Pga7 at close to 1∶1 concentration.

Both recombinant proteins specifically bound to a hemin-agarose column (Fig. 5A). Binding was stable in high salt and at acidic pH, but some release occurred at neutral and alkaline pH. Imidazole serves, within the side chain of histidine, as a proximal (and sometimes distal) ligand of heme iron in many heme proteins [28]; high imidazole concentrations achieved almost complete release of both proteins from the hemin beads (Fig. S4). To confirm the importance of the conserved CFEM domain in heme binding, we mutagenized an aspartic residue conserved in most CFEM domain proteins [15] (Asp63 of Pga7 and Asp72 of Rbt5; Fig. S1) to alanine, and tested binding of the mutants to the hemin column. Binding of the mutant proteins to the column was essentially abolished, confirming the role of the CFEM domains of Pga7 and Rbt5 in heme binding (Fig. 5A).

To more quantitatively compare the heme binding properties of Pga7 and Rbt5, we used isothermal calorimetry (ITC) to measure the stoichiometry of binding and the affinity of Rbt5_23–219_ and Pga7_18–195_ for hemin (Fig. 5B). Average fitting of three independent experiments on different batches of the proteins gave an affinity (*K_D_*) of Pga7_18–195_ and Rbt5_23–219_ for heme of 6.5×10^−8^ M and 5.4×10^−6^ M, respectively. Thus, the affinity of Pga7 towards heme is ∼100-fold higher than that of Rbt5. Saturation for both proteins was reached at a molar ratio of about two heme per protein molecule. However, rather than indicating two binding sites per protein molecule, this stoichiometry could be explained by the fact that for the ITC experiments, the proteins were titrated into 10 µM or 20 µM hemin solutions, and that at neutral pH, at concentrations over 10 µM, heme is almost exclusively (>90%) dimeric [29].

To investigate the stoichiometry further, we used the shift in absorbance of heme from 380–390 nm to a sharp 406 nm peak upon binding to a protein (Soret bandshift) as an alternative method for assaying heme-protein interaction. Hemin was titrated into an apoPga7 solution, keeping the free hemin at sub-micromolar levels, and absorbance at 406 nm was recorded. Addition of heme beyond saturation of Pga7 is expected to shift the absorption maximum towards 390 nm, representing the unbound heme. To obtain the concentration at which Pga7 becomes saturated with heme, the relative absorbance of Pga7-heme to heme alone at 406 nm was plotted against hemin concentration. The inflection point indicated that saturation of the protein (5.5 µM apoPga7) was reached at 5.1 µM of heme (Fig. 5C), consistent with a 1∶1 heme to Pga7 stoichiometry, suggesting that Pga7 contains a single heme binding site.

**Figure 6:**
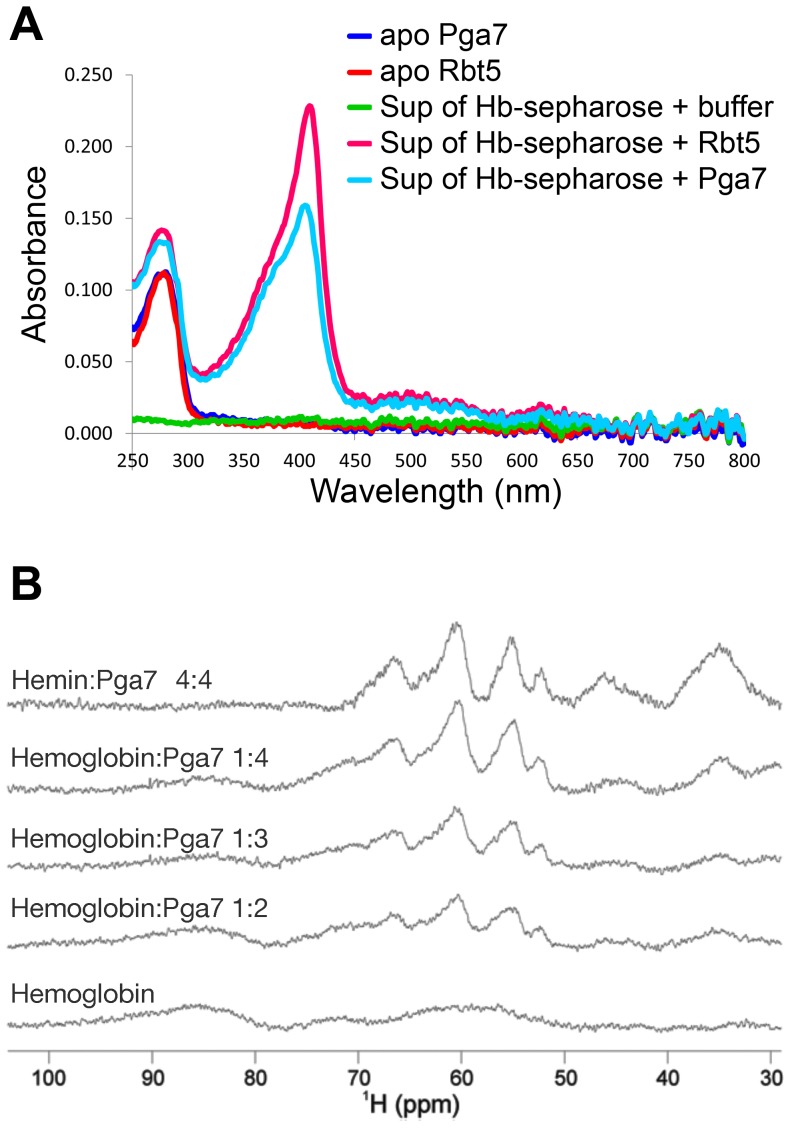
Pga7 and Rbt5 can extract heme from hemoglobin. (A) Hemoglobin covalently conjugated to CnBr sepharose beads was incubated with 50 µM recombinant Rbt5_23–219_ or Pga7_18–195_, or with PBS buffer alone. After 30 min incubation, the UV-Vis spectrum of the supernatant was measured with a Nano-Drop 2000 spectrophotometer. The peak at 280 nm represents the protein. The 406 nm Soret absorption peak visible in the supernatants containing Rbt5 and Pga7 is typical of protein-bound heme. The average reading of a triplicate experiment is represented. (B) Upfield areas of ^1^H NMR spectra recorded at 600 MHz and 298 K to monitor the transfer of heme to Pga7_18–195_. Pga7 shows signals attributable to heme methyls in the range 53–67 nnm (top panel). Hemoglobin is characterized by broader unresolved features in the 50–100 ppm range. Titration of hemoglobin (middle panels) with increasing amounts of Pga7 gives rise to the transfer of the heme from hemoglobin to Pga7 with progressive disappearance of the broad features of hemoglobin and appearance of the characteristic signals of heme methyls in Pga7. Note that hemoglobin contains 4 heme groups per molecule.

Heme binding to Pga7 was further confirmed by monodimensional ^1^H nuclear magnetic resonance (NMR) titrations where the formation of the heme-Pga7 adduct was followed through the appearance of signals for peripheral methyls of the porphyrin ring in the 53–67 ppm range (see below, Fig. 6B), which are diagnostic for the formation of the protein-iron(III) heme complex [30], [31].

### Rbt5 and Pga7 can extract heme from hemoglobin *in vitro*

The involvement of Rbt5 and Pga7 in hemoglobin utilization, and the slightly increased growth defect of the *pga7^−/−^rbt5^−/−^* mutant over *pga7^−/−^* alone in the presence of hemoglobin but not of hemin (Fig. 1 and S2) led us to investigate the interaction of recombinant Pga7 and Rbt5 with hemoglobin. A sepharose column with covalently bound hemoglobin was incubated with Pga7_18–195_ or Rbt5_23–219_, and the supernatants were separated from the beads. No stable binding of either Rbt5 or Pga7 to the hemoglobin beads could be detected. However, the supernatants containing either protein, but not buffer alone, exhibited a faint yellow color. To test whether this color represents heme bound to the CFEM proteins, visible light spectrophotometry was used to detect the characteristic Soret peak at 406 nm, representing heme incorporated into the protein. As shown in Fig. 6A, both Rbt5 and Pga7 acquired the Soret peak after incubation with the hemoglobin column. The heme was not spontaneously released from the globin, as shown by the lack of absorbance at 380–390 nm in the “buffer alone” extract, and no globin proteins were detected by Western blotting in any of the extracts, indicating that Pga7 and Rbt5 did not cause dissociation of the hemoglobin from the column. We thus concluded that Rbt5 and Pga7 are both able to extract heme from hemoglobin.

The hemoglobin heme extraction activity of Pga7 was more surprising than that of Rbt5, as the *pga7^−/−^* mutant did not exhibit any hemoglobin-specific growth defect (Fig. 1 and S2) and as Pga7 is buried in the cell envelope. To confirm the ability of Pga7 to extract heme from hemoglobin, we used monodimensional ^1^H NMR as an alternative method, and took advantage of the differences in heme methyl resonances of heme bound to globin vs. to Pga7. Hemoglobin displays three broad unresolved features centered around 64, 72 and 86 ppm (Fig. 6B, bottom), whereas heme-Pga7 shows four peaks around 53, 55, 62 and 67 ppm (Fig. 6B, top). Upon titration of hemoglobin with apo-Pga7, the pattern of heme-Pga7 chemical shifts gradually appeared, whereas the hemoglobin chemical shifts disappeared upon reaching a 4∶1 Pga7:hemoglobin stoichiometry (Fig. 6B). At the same time, a white precipitate formed, probably representing the less-soluble apoglobin [32]. These data are consistent with efficient extraction of heme from hemoglobin by Pga7 in solution.

### Heme transfer between Rbt5 and Pga7

**Figure 7:**
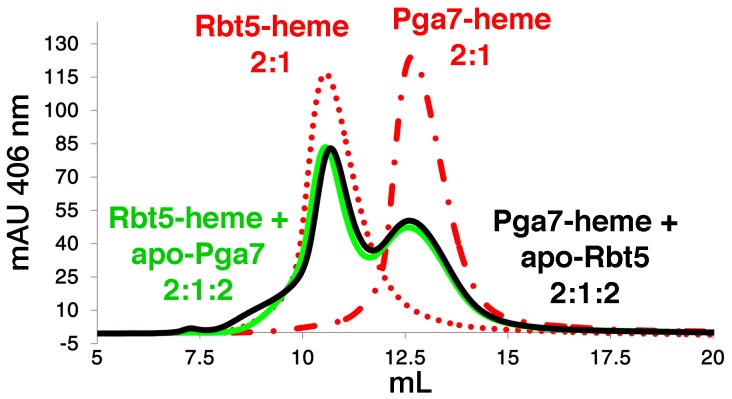
Transfer of heme between Rbt5 and Pga7. The indicated proteins were separated by gel filtration and the protein-bound heme was detected by absorbance at 406 nm. Red curves: 50 µM of either apo-Rbt5_23–219_ or apo-Pga7_18–195_ in 100 µl was incubated for 5 min with 25 µM heme before loading on the column. Black and green curves: after pre-incubation of apo-Rbt5_23–219_ or apo-Pga7_18–195_ with heme, 50 µM of the second protein was added and incubated a further 5 min prior to loading on the column. The areas under the curves were similar (+/−10%) in all four runs.

The different localization and different affinities to heme of Rbt5 and Pga7 raised the possibility of a relay system, in which heme is transferred from one protein to the next across the cell wall before being endocytosed. To test whether heme could indeed be transferred directly between Rbt5 and Pga7, we used a size exclusion chromatography column that efficiently separates both proteins. Absorbance at 280 nm was used to monitor the proteins, and absorbance at 406 nm indicated protein-bound heme. The initial protein preparations were confirmed to be free of heme (Fig. S5, top panels). Rbt5 and Pga7 were then each incubated with sub-stoichiometric amounts of heme (2∶1) and separated on the chromatography column: the heme peaks (406 nm) coincided with the protein peaks (280 nm) for each protein, indicating stable heme binding to the proteins throughout the chromatography run (Fig. S5, bottom panels). Next, following pre-incubation of Rbt5 with heme, the same amount of Pga7 was added to the mix, and after another 5 min incubation, the mix was injected to the column. Separation by size exclusion revealed a redistribution of the total heme amount between the two protein peaks, indicating that heme is partially transferred from Rbt5 to Pga7 (Fig. 7). In the reciprocal experiment, where Pga7 pre-loaded with heme was mixed with Rbt5, an identical final redistribution of the total heme amount between both proteins was obtained, indicating that the redistribution had reached equilibrium (Fig. 7). The total amount of heme was identical, within 10%, in all cases, indicating that the appearance of a signal corresponding to the second protein is not due to binding of free heme, but must instead represent transfer of heme from one protein the other.

**Figure 8:**
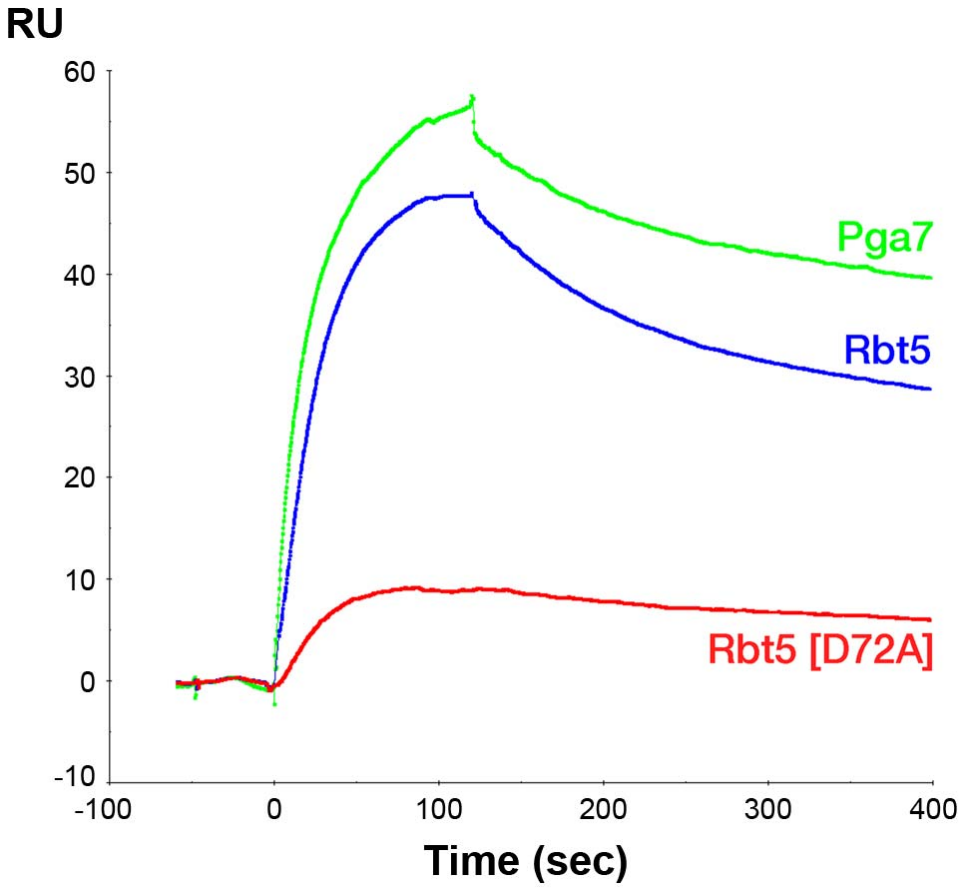
Holo-Pga7 interacts with apo-Rbt5 and with apo-Pga7. SPR analysis was carried out by immobilizing apo-Pga7_18–195_, apo-Rbt5_23–219_, and the D72A mutant of apo-Rbt5_23–219_, on a Biacore biosensor chip. 3 µM holo-Pga7_18–195_ was injected at time zero for 60 sec.

Since the affinity of Pga7 for heme is much higher than that of Rbt5, the approximately equal redistribution of heme between the two proteins is not consistent with a heme release-and-capture transfer mechanism between the proteins, but rather is expected to involve a contact between Pga7 and Rbt5, either direct, or mediated by heme. In order to test this, we measured homologous and heterologous interactions between the proteins by surface plasmon resonance (SPR), both in the presence and absence of heme. Recombinant Pga7, Rbt5, and the Rbt5 D72A mutant, which is defective in heme binding (Fig. 5), were bound to the biosensor, and interaction with holo- or apo-Pga7 was measured. Holo-Pga7 interacted with both Rbt5 and Pga7, but much more weakly with the Rbt5 D72A mutant (Fig. 8). The almost complete lack of interaction with the mutant protein serves as control for the specificity of the interaction of holo-Pga7 with the bound wild-type proteins. Apo-Pga7 exhibited an interaction with apo-Rbt5, but not with apo-Pga7, nor with the apo-Rbt5 D72A mutant (Fig. S6). These data are consistent with a heme transfer mechanism between Pga7 and Rbt5 that involves interaction between the proteins. Furthermore, the heme-induced interaction detected between Pga7 bound to the biosensor chip and in solution suggest that heme can be transferred between homologous proteins as well.

## Discussion

While small nutrient molecules are assumed to freely diffuse across the cell wall matrix, the cell wall represents a permeability barrier for larger molecules. The molecular weight limit for diffusion through the fungal cell wall was found to vary greatly across different studies, with thresholds as low as 620 Da [33] and as high as several hundred kDa [34] (heme is 616 Da). Some of the discrepancies in porosity may be due to differences in yeast species used, growth medium [34], [35], or growth phase – with reduced porosity in stationary phase [36] (reviewed in [22], [37]). Even in individual growing cells, porosity is probably not uniform, and it is likely that mature cell wall is less porous than the newly synthesized cell wall at the bud or hyphal tip of growing cells, and may thus require a system for transporting even relatively small molecules. In analyzing the pathway of heme-iron uptake across the cell envelope of the pathogenic yeast *C. albicans*, we found that utilization of both free heme and hemoglobin requires a network of at least two distinct cell surface proteins of the CFEM family, Rbt5 and Pga7. This network may represent the first instance of a cell wall nutrient carrier system in yeast. Other fungi also express heme transport systems, notably *Cryptococcus neoformans*, *Candida parapsilosis*, and *Paracoccidioides brasiliensis*. *P. brasiliensis* and *C. parapsilosis* were shown to rely, like *C. albicans*, on CFEM proteins for heme-iron utilization [38], [39], whereas *C. neoformans* relies on an unrelated heme-binding protein, Cig1, for heme-iron utilization [40]. However the mechanism by which these proteins mediate heme transfer across the cell wall and internalization into the cell is unknown.

While investigating the reason for the requirement for two apparently similar proteins under a given set of conditions, we found that Rbt5 and Pga7 differ in localization, abundance, and affinity for heme. Immunolocalization of Rbt5 and Pga7 indicated that Rbt5 is more exposed, as it is detectable by specific antibodies at the cell surface of untreated cells, whereas Pga7 is not. Interestingly, Rbt5 was identified as one of the top *C. albicans* antigens recognized by antisera of patients recovering from candidemia [41], suggesting that Rbt5 is preferentially presented to the host's immune system during infection, consistent with an exposed localization on the cell envelope.

The observation that both Pga7 and Rbt5 are able to efficiently extract heme from hemoglobin *in vitro* reveals a new function for CFEM proteins and suggests a mechanism whereby the heme is extracted from hemoglobin in the cell wall, and is transferred to one of these two proteins. The expected difficulty for the large hemoglobin molecules to diffuse through the cell wall matrix, and the peripheral location of Rbt5 as compared to Pga7, would predict that Rbt5 plays a larger role in hemoglobin than in hemin iron utilization. and might thus explain why in the absence of Pga7, the presence of Rbt5 was found to slightly ameliorate hemoglobin utilization, but not hemin utilization (Figs. 1 and S2). This model, involving extraction of heme from hemoglobin at the cell periphery, followed by internalization of heme into the cell, goes against our previous observation that fluorescently tagged hemoglobin is taken up into the vacuole in an Rbt5-dependent manner [18]. To reconcile between the observations, we suggest that either hemin can also be labeled with rhodamine via a non-conventional reaction, or that after heme extraction, the tagged globin molecules are taken up by fluid-phase endocytosis into the vacuole.

**Figure 9:**
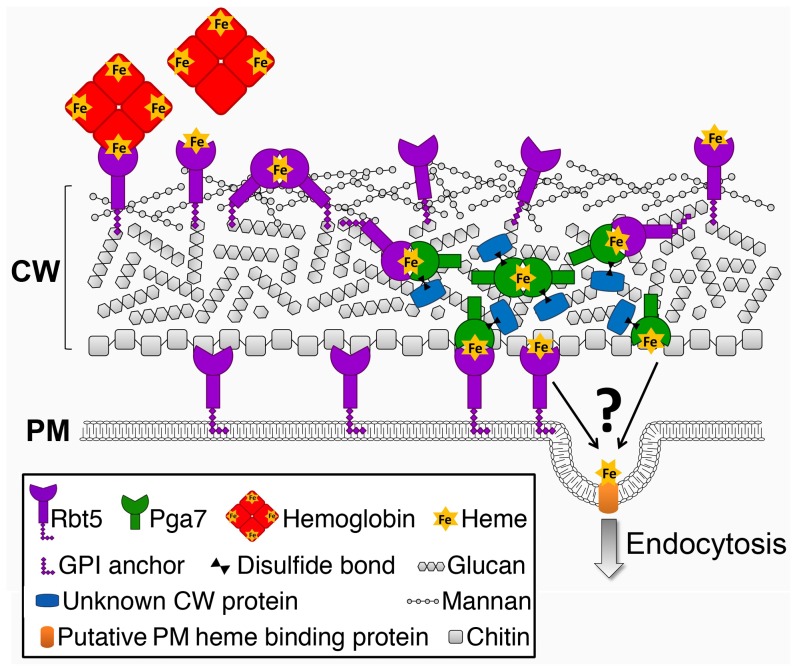
Schematic model of the heme relay network that extracts heme from hemoglobin and delivers it to the endocytic pathway. Cell wall polysaccharide organization was modeled after [62], [63]. The identity of the membrane receptor that mediates internalization into the cell – Rbt5, Pga7, or another protein – is unknown.

Cellular nutrient uptake typically occurs via plasma membrane transporters. The stable binding of heme to the cell wall proteins Pga7 and Rbt5 generates a conundrum: how can stable binding of a ligand to a protein embedded in the cell wall matrix result in uptake of the ligand into the cell? Rbt5 is found to be attached mainly via an HF-sensitive linkage to the cell wall, indicative of a covalent linkage to the end of β-(1,6)-glucan chains, as opposed to Pga7, which is less abundant, appears by immunodetection to be buried within the cell envelope, and displays fewer HF-sensitive linkages and more β-ME -sensitive linkages to the cell wall. The location of a protein at the end of a hydrophilic β-(1,6)-glucan chain was suggested to confer a certain amount of diffusional freedom, constrained only by the length of the chain [23]. The abundance of Rbt5 was estimated to be on the order of 10^6^ molecules per cell, based on quantitation of Rbt5-dependent ^55^Fe-heme binding to *C. albicans* cells [14], suggesting that Rbt5 molecules could be as close as 10 nm apart on average on the surface of a 5 µm-wide cell. It is thus conceivable that the diffusional freedom conferred by the β-(1,6)-glucan anchor would enable Rbt5 molecules to interact with one another. We found that heme could be rapidly exchanged between Rbt5 and Pga7 *in vitro*. The observation that heme can induce an homologous interaction between Pga7 molecules (Fig. 8) suggests that it can be transferred between homologous proteins as well, which would enable it to be channeled from one CFEM protein molecule to the next until it reaches the plasma membrane (Fig. 9). Such a relay system would thus explain how heme-binding proteins embedded in the rigid cell wall matrix can mediate heme uptake rather than serving as heme sinks.

The ability of the heme to be efficiently transferred from one CFEM molecule to the next within a single cell's wall also raises the possibility that, given sufficient proximity, or in the presence of a hemophore, the heme could similarly be transferred between different cells. Notably, recent results indicating that the secreted CFEM protein Csa2 also plays a role in heme-iron utilization ([42], [43] and our unpublished results), suggest that the CFEM heme-transfer cascade in fact includes a soluble hemophore. Such a molecule might conceivably be able to transfer heme not only from the environment to the cell, but also between adjacent cells, such as occur e.g. within biofilms, and it is thus notable that CFEM proteins were found to play a role in *C. albicans* biofilm formation [44].

Although we did not detect directionality in the transfer of heme between Rbt5 and Pga7, directionality of the flow of heme may be conferred by the irreversibility of the endocytosis step once the heme molecules have reached the plasma membrane. The endocytic pathway was found to play an essential role in heme utilization [18]. A possible role for Pga7 would be to deliver heme to the endocytic apparatus. Whether this would entail another, third heme-binding component, or whether membrane-bound fractions of Rbt5 or Pga7 accompany heme through endocytosis, is not known. Genetic epistasis analysis of the *rbt5^−/−^* and *pga7^−/−^* mutants indicated that *pga7^−/−^* is more defective in heme utilization, and that it is epistatic to *rbt5^−/−^*. The model for heme uptake proposed above can readily explain this genetic relationship: whereas Pga7 would function in helping heme to penetrate the cell via endocytosis, Rbt5 would merely facilitate diffusion of heme across the cell wall. Thus, in the absence of Rbt5, heme utilization is limited by the extent of free diffusion of heme or hemoglobin through the outer layers of cell wall, while in the absence of Pga7, its utilization may be limited by the more restricted ability of the cell to internalize heme from the external medium by general fluid-phase endocytosis. An alternative explanation for the differential phenotype of *rbt5^−/−^* vs. *pga7^−/−^* is that the higher affinity of Pga7 for heme is responsible for its requirement at lower heme concentrations. Pga7 and Rbt5 might thus represent independent high-affinity, low-throughput and a low-affinity, high-throughput systems of heme uptake, respectively, similar perhaps to the parrallel high- and low-affinity uptake systems for e.g. ammonium [45] or iron [46]. However in view of the observed transfer of heme between Rbt5 and Pga7, of the coordinate regulation of the two genes, and of their different cellular location, we favor the model that places both proteins within a single sequential heme uptake system.

CFEM proteins are found in many fungi. Alignment of the CFEM proteins sequences most similar to Rbt5 and Pga7 that were identified in 6 fungal genomes reveals clearly distinguishable Rbt5 and Pga7 branches (Fig. S7). Each of six fungal genomes screened appears to carry at least one Rbt5 homolog and one Pga7 homolog, possibly mirroring the functional distinction between these proteins in heme uptake, and suggesting that the relay network of heme acquisition may be conserved in these other fungi. It is not clear however that all CFEM proteins are involved in heme uptake, since *S. cerevisiae*, which does not utilize heme-iron [8], does express a CFEM protein, Ccw14/Icwp, and since none of the 3 *Aspergillus fumigatus* CFEM proteins could be shown to bind hemin or to promote heme utilization [47]. On the other hand, *Candida glabrata CCW14*, the apparent ortholog of *C. albicans SSR1*, was recently shown to affect intracellular iron homeostasis [48], suggesting a role in iron uptake of this class of CFEM proteins as well. In addition, common to the mutants in all the CFEM genes analyzed to date, be they members of the Rbt5/Pga7 clade in *C. albicans*, the *A. fumigatus* CFEM proteins, or *S. cerevisiae* Ccw14, are phenotypes consistent with structural defects in the cell wall ([47], [49], [50] and our unpublished data). The notion that Rbt5 and Pga7 are part of a dynamic protein network involved in heme transfer does not exclude the possibility that regardless of their nutrient uptake activity, CFEM proteins are able to form structural networks of proteins that contribute to the rigidity of the yeast cell wall.

*C. albicans* is a commensal organism of the human digestive system, and it is presumably in this context that the heme utilization pathway normally functions. The estimated one-third of iron ingested as heme-iron in a typical Western diet is indeed relatively more bioavailable, compared to non-heme iron, in the alkaline conditions of the small intestine lumen [51], and mouse gut-adapted *C. albicans* cells, while exhibiting reduced expression of elemental iron uptake genes, show persistent expression of heme uptake pathway genes [52]. Nonetheless, we find that heme-iron utilization contributes to the virulence of *C. albicans* in a systemic infection model, indicating that this pathway may play a role in the context of a systemic infection as well. The prognosis of systemic candidiasis is still poor, with a crude mortality rate of 40% [53]. The identification of the network of cell surface proteins involved in heme-iron uptake, and the demonstration that this pathway is important for the virulence of *C. alicans* in a mouse bloodstream infection model, may open up new possibilities for inhibiting this pathogen, either by interfering with the CFEM heme uptake relay network, or by taking advantage of this network to specifically introduce toxic compounds into the cell.

## Materials and Methods

### Ethics statement

All experiments were performed under strict adherence to the National Institutes of Health guidelines for the ethical treatment of animals. Signs of infection (torticollis, lethargy, ataxia) were monitored three times daily throughout the experimental time course. Moribund mice were euthanized by cervical dislocation under isofluorane anesthesia. Approval for these studies was granted by the University of Tennessee Animal Care and Use Committee on September 7, 2011 until September 6, 2014 (protocol # 016-0911). The University of Tennessee has an Animal Welfare Assurance on file with The Public Health Service and is under regulation of the USDA and PHS. UT Animal Welfare Assurance Number – A3668-01. UT AAALAC Accreditation File Number – File 000178 (UTK is fully accredited by AAALAC).

### Media and growth conditions

*C. albicans* strains were grown in rich medium (YPD; 1% yeast extract, 2% Bacto peptone, 2% glucose, 25 µg/ml uridine). Transformants were selected on SC-URA plates (0.17% yeast nitrogen base; 0.5% ammonium sulphate, 2% glucose, 2% agar). For iron starvation, cells were grown in iron poor medium (IPM) or in iron free medium (IFM). IPM was prepared by supplementing YPD with 1 mM ferrozine (Sigma) [8], [54] IFM was prepared as described previously [8], [18] with the following modifications: RPMI-1640 HEPES (Sigma) was supplemented with 2% glucose, 25 µg/ml uridine and 25 µg/ml adenine, then the medium was desferrated by 5% w/v Chelex100 (Sigma) for 2 hours and 100 µg/ml human serum iron chelator apotransferrin (Sigma) was added. Chelex100 beads were removed by filtration and essential metals except iron were restored (40 µg/L CuSO_4_, 400 µg/L ZnSO_4_, 400 µg/L MnCl_2_, 200 µg/L MoNa_2_O_4_ and 200 mg/L CaCl_2_). The final pH was adjusted to 7.2 with 1N HCl, and only medium prepared the same day was used.

*P. pastoris* was grown in YPD. Transformants were selected on YPD supplemented with 100 or 1000 µg/ml Zeocin (Invitrogen). Buffered Glycerol-complex Medium (BMGY; 1% yeast extract, 2% peptone, 100 mM potassium phosphate, pH 6.0, 1.34% YNB, 4×10^−5^% biotin, and 1% glycerol) and Buffered Methanol-complex Medium (BMMY; 1% yeast extract, 2% peptone, 100 mM potassium phosphate, pH 6.0, 1.34% YNB, 4×10^−5^% biotin, and 0.5% methanol) were used for protein expression.

### Hemin and hemoglobin preparation

All stocks were freshly prepared before each assay. For growth assays, stock solutions of 0.1 mM human hemoglobin (Sigma) in PBS, 0.5 mM bovine hemoglobin (Sigma) in PBS, or 2 mM hemin (Frontier Scientific) in 0.2 N NaOH were chelated with 5% w/v Chelex100 (Sigma) for 2 hours before use. For all other assays, hemin was prepared in PBS in the presence of 10 mM NaOH. For NMR analysis, stock solutions of 0.1 mM bovine hemoglobin in PBS and of 25 mM hemin in 0.1 N NaOH were used.

### Plasmids and strains

Ca*PGA7* 5′ region (−930)-(−1) and 3′ region (+661)-(+1100) as SacI-SpeI and HindIII-KpnI PCR fragments, respectively, were cloned into KB986 [55], containing the hisG-URA3-hisG fragment, to generate the *PGA7* deletion plasmid (KB2025). A *PGA7* complementation plasmid (KB2111) was generated by cloning the *PGA7* region from (−570) to (+1343) as a HindIII-KpnI PCR fragment into pBES116 [56]. The FLAG tag coding sequence was introduced on a primer and fused after the signal sequence, at position +52 of *PGA7* and +79 of *RBT5*, and introduced into pBES116 as HindIII-KpnI fragments to generate KB2189 and KB2268, respectively. To overexpress FLAG-tagged Pga7 under iron starvation, the RBT5 promoter region (−550)-(−1) and the FLAG-tagged PGA7 open reading frame from KB2189 were fused by PCR and inserted as HindIII-KpnI fragments into pBES116 to generate KB2274. To generate Pichia-expressed proteins lacking signal peptide and GPI anchor consensus sequences, KB2259 and KB2258 were constructed by amplifying *PGA7* (+52) to (+585) and *RBT5* (+67) to (+657), respectively, and introducing them as EcoRI-XbaI into pPICZαA (Invitrogen). The aspartate→alanine mutants were constructed by PCR-mediated site directed mutagenesis of KB2259 and KB2258 to generate KB2296 and KB2271, respectively.

*C. albicans* strains are listed in Table 1. Deletion of *PGA7* was obtained in CAI-4 or CAF3-1 background by sequential deletion of both alleles using plasmid KB2025 [57]. The deletions were confirmed by PCR and Southern blot. *Pichia pastoris* strains for expression of recombinant proteins are listed in Table 2. SacI-linearized plasmids (KB2258, KB2271, KB2259, KB2258, or KB2156) were integrated at the *AOX1* locus of *P. pastoris* X-33 (Invitrogen) using the LiCl method as described in EasySelect *Pichia* Expression Kit (Invitrogen), except that after four hours recovery, 100 µl were plated on YPD +100 µg/ml Zeocin, and the rest of the cells were plated on YPD +1 mg/ml Zeocin.

**Table 1:** List of *C. albicans* strains.

Name	Genotype or description	Origin
KC2 = CAF3-1	*ura3Δ::imm434/ura3Δ::imm434*	[57]
KC78 = CAI4	*ura3Δ::imm434/ura3Δ::imm434*	[57](*via* A. Johnson)
KC590 = CAI4	*ura3Δ::imm434/ura3Δ::imm434*	[57](*via* J. Becker)
KC482	KC2 *pga7Δ::hisG/pga7Δ::hisG*	This study
KC605	KC482 *ade2:: ^FLAG^PGA7* Ca*URA3*	This study
KC712	KC482 *ade2::RBT5p- ^FLAG^PGA7* Ca*URA3*	This study
KC645	KC590 *ade2::*Ca*URA3*	This study
KC626	KC590 *pga7Δ::hisG/pga7Δ::hisG*	This study
KC646	KC626 *ade2::*Ca*URA3*	This study
KC647	KC626 *ade2::*Ca*URA3 PGA7*	This study
KC589	CAI-4 *rbt5Δ::hisG/rbt5Δ::hisG*	This study
KC706	KC589 *ade2:: ^FLAG^RBT5* Ca*URA3*	This study
KC594	KC589 *pga7Δ::hisG/pga7Δ::hisG*	This study
KC68	KC2 Ca*ccc2Δ::hisG/*Ca*ccc2Δ::hisG*	[8]
KC811	KC68 *ade2::*Ca*URA3*	This study
KC485	KC68 *pga7Δ::hisG/pga7Δ::hisG*	This study
KC684	KC485 *ade2::*Ca*URA3*	This study
KC536	KC485 *ade2::*Ca*URA3-PGA7*	This study
KC617	KC485 *ade2::*Ca*URA3 ^FLAG^PGA7*	This study
KC711	KC485 *ade2::* Ca*URA3 RBT5p- ^FLAG^PGA7*	This study
KC139	CAI-4 Ca*ccc2Δ::hisG/*Ca*ccc2Δ::hisG rbt5Δ::hisG/rbt5Δ::hisG*	This study
KC683	KC139 *ade2::*Ca*URA3*	This study
KC621	KC139 *ade2:: RBT5* Ca*URA3*	This study
KC713	KC139 *ade2:: ^FLAG^RBT5* Ca*URA3*	This study
KC488	KC139 *pga7Δ::hisG/pga7Δ::hisG*	This study
KC170	CAI-4 *rbt5Δ::hisG/rbt5Δ::hisG rbt51Δ::hisG/rbt51Δ::hisG*	This study
	Ca*ccc2Δ::hisG/*Ca*ccc2Δ::hisG csa1Δ::hisG/csa1Δ::hisG*	
KC508	KC170 *pga7Δ::hisG/pga7Δ::hisG*	This study

**Table 2:** List of *P. pastoris* strains.

Name	Genotype or description	Origin
KC755	Mut^+^ Zeo^R^ Pga7(18–195a.a.)- Myc-6×His	This study
KC756	Mut^+^ Zeo^R^ Pga7(18–195a.a.) D63A-Myc-6×His	This study
KC758	Mut^+^ Zeo^R^ Rbt5(23–219a.a.)-Myc-6×His	This study
KC759	Mut^+^ Zeo^R^ Rbt5(23–219a.a)D72A-Myc-6×His	This study

### Growth assays

Overnight cultures grown in YPD were diluted in the morning into a series of two-fold dilutions of hemin or hemoglobin in IFM or IPM. Cells were inoculated in flat-bottomed 96-well plates at OD_600_ = 0.0002 for IFM or OD_600_ = 0.000005 for IPM. Plates were incubated at 30°C with 60 rpm shaking. Growth was measured as optical density (OD_600_) with an ELISA reader after 3 days.

### Virulence assay

The parental strain was compared to the *pga7^−/−^* mutant strain and the re-integrant strain. The *PGA7* gene was reintroduced in the *C. albicans* genome on a *URA3*-marked plasmid. Since the *URA3* presence and position of integration into the genome is known to affect the virulence of *C. albicans* [58], [59], we introduced the *URA3* vector into the WT strain and the *pga7^−/−^* mutant strain at the same *ADE2* genomic location. *C. albicans* strains were grown overnight in 50 ml of YPD at 30°C with constant shaking. The strains were harvested, washed twice with water, and resuspended to 1×10^7^ cells/ml in water. Each strain was injected via tail vein into a group of 5 mice, 0.1 ml suspension (1×10^6^ cells) per mouse. Mice were housed five per cage with food and water supplied ad libitum. Signs of infection (torticollis, lethargy, ataxia) were monitored three times daily throughout the experimental time course. Moribund mice were euthanized by cervical dislocation under isofluorane anesthesia. Survival fractions in virulence tests were calculated by the Kaplan-Meier method, and survival curves were tested for significant difference (P<0.01) by the Mantel-Haenszel test using GraphPad Prism.

### Pulse-labeling

To measure the expression of FLAG-tagged proteins under iron starvation conditions, *C. albicans* cells were grown overnight in IPM at 30°C, diluted 1∶20 in the same medium, and grown 2 hours to OD_600_ = 1.3. 1.5 ml of the culture were spun down, washed twice in low-iron YNB +1 mM ferrozine, resuspended in 0.1 ml of this medium supplemented with 0.25 mCi ^35^S methionine/cysteine (Perkin Elmer Easytag Express) and incubated for 5 min at 30°C. Proteins were then extracted and immunoprecipitated as described in [60], using a rabbit anti-FLAG antiserum Proteins were separated by SDS-PAGE and visualized and quantitated using a GE Typhoon FLA 7000 phosphorimager.

### Protein localization

Samples for protein localization by immunofluorescence and cell fractionation were taken from a single initial culture in each experiment. To obtain maximal levels of expression, and given that *RBT5* and *PGA7* are both induced by iron starvation, *ccc2^−/−^* background strains were used, which are defective in high-affinity iron uptake, and therefore partially iron starved even in regular culture medium. Overnight cultures in YPD medium were extensively washed with water, inoculated in iron-poor medium at OD_600_ = 0.3, and grown to OD_600_ = 4 (about 6 hours). For immunofluorescence, 5 ml aliquots were fixed with 4.5% formaldehyde in 1×PBS for 20 min and then subjected to the immunofluorescence detection protocols, as described below. A second 5 ml aliquot from the same culture was harvested by centrifugation, washed with cold water and with 50 mM Tris pH 7.5, snap-frozen in liquid nitrogen and stored at −80°C, before sequential extraction (see below).

Immunofluorescence microscopy: untreated cells were rinsed once in PBS +0.1% bovine serum albumin (PBS-BSA), then blocked for 1 hour with PBS-BSA before overnight incubation at 4°C in PBS-BSA containing 1∶500 monoclonal anti-FLAG M2 antibody (Sigma). Cells were then extensively washed with PBS-BSA, and incubated in PBS-BSA containing Cy3-conjugated Donkey anti-mouse IgG at 1∶200 dilution (Jackson ImmunoResearch) for 1 hour at room temperature. Cells were then washed 5 times with PBS and placed on polylysine-coated slides. Samples fixed on slides were rinsed once with PBS before mounting in Vectashield mounting medium containg DAPI (Vector Laboratories). NaOH-treated cells were resuspended in 250 mM NaOH and incubated for 20 minutes on ice before proceeding with antibody staining as described above. For cell permeabilization, cells were washed with phosphate buffer (pH 7.5), then resuspended in sorbitol buffer (phosphate buffer pH 7.5 containing 1.2 M sorbitol) with 0.5 mg/ml Zymolyase 100T (USBiological) and 0.1% β-ME in sorbitol buffer, and incubated for 10 minutes at 37°C. Two volumes of cold sorbitol buffer were added before centrifugation and 2 additional washes in sorbitol buffer. Cells were placed on polylysine-coated slides, then blocked in PBS-BSA as above, and incubated in the presence of 1∶500 monoclonal anti-FLAG M2 antibody (Sigma) in PBS-BSA overnight in a humid chamber at 4°C. Slides were washed with PBS-BSA and incubated with Cy3-conjugated Donkey anti-mouse IgG (Jackson ImmunoResearch) for 1 h at room temperature, rinsed once with PBS-BSA, and mounted with Vectashield + DAPI (Vector Laboratories). Epifluorescence microscopy was performed using a Zeiss AxioImager M1 equipped with a plan- Apochromat 100× oil immersion objective and rhodamine and DAPI filter sets.

For sequential cell extraction, the frozen 5 ml cell culture pellets were resuspended in 50 mM Tris-HCl, pH 7.5, and mechanically broken with 425–600 µm glass beads (Sigma) in the presence of a protease inhibitor cocktail. (1 mM phenylmethylsulfonyl fluoride (PMSF), and 0.02 mg/ml each of chymostatin, pepstatin A, leupeptin, and antipain), in a BeadBeater apparatus for 4 pulses of 1.5 min each with 5 min intervals on ice. To remove the cytosolic proteins, the lysate was centrifuged in an Eppendorf centrifuge at max speed at 4°C for 30 min, and the supernatant was removed and concentrated to 200 µl using an Amicon Ultra 3K MWCO filter (Millipore). The pellets were then washed extensively with 1M NaCl, and the wash fractions were pooled and concentrated to 200 µl in an Amicon Ultra 3K MWCO filter. To remove membrane proteins, the pellets were extracted 5 times by heating 5 min at 95°C in 50 mM Tris pH 7.5, 2% SDS, centrifuged at RT, and the supernatants were pooled and concentrated. To remove disulfide-linked cell wall proteins, the pellets were extracted 4 times by heating 5 min at 95°C in 50 mM Tris pH 7.5, 2% SDS, and 4% β-ME, centrifuged at RT, and the supernatants were pooled and concentrated. The remaining pellets were extensively washed with cold water, dried and frozen in liquid nitrogen. To release GPI-ß1,6-glucan-linked cell wall proteins, frozen cell wall pellets were incubated in 200 µl HF-pyridine (Sigma) at 0°C for 3 h [23]. The supernatants were removed after centrifugation and precipitated by addition of 9 volumes of 95% methanol buffer (95% [vol/vol] methanol, 5% 1 M Tris-HCl pH = 7.5) and incubated at 0°C for 2 hours. The pellets were washed 3 times with 85% methanol buffer (85% methanol, 5% 1 M Tris-HCl pH = 7.5, 10% H_2_O), dried and resuspended in 2× Laemmli Sample Buffer. 1/50 of each fraction was loaded onto precast 4–20% TGX gels (BioRad), followed by Western blot transfer to Immobilon transfer membranes (Millipore). Blots were reacted with a polyclonal anti-FLAG antibody (Sigma; 1∶1,000) and secondary IRDye 800 antibody (LI-COR; 1∶10,000), followed by imaging and quantitation of the FLAG-tagged proteins using the Odyssey Infrared Imaging System (LI-COR).

For detection of FLAG-Pga7 and Rbt5 at native expression levels, an overnight culture in YPD was washed once with water and diluted to OD_600_ = 0.3 in 10 ml IFM, then grown for 5 hours to OD_600_ = 2. The cells were then harvested by centrifugation, resuspended in 50 mM Tris-HCl, pH 7.5, and mechanically lysed using a bead-beater apparatus. Cell fractionation was carried out as described above but the lysate supernatant and NaCl extractions were discarded. The membrane proteins were extracted once by heating for 5 min at 100°C in 50 mM Tris pH 7.5, 4% SDS, centrifuged at RT, and the disulfide-linked cell wall proteins were extracted once by heating for 5 min at 100°C in 50 mM Tris pH 7.5, 4% SDS, and 4% β-mercaptoethanol, followed by centrifugation at RT. 1/5 of each fraction was loaded onto a 6%–15% gradient SDS-PAGE gel, followed by Western blot transfer to a nitrocellulose membrane. Blots were incubated with monoclonal anti-FLAG M2 antibody (Sigma; 1∶1,000) and secondary anti-mouse-HRP antibody (Jackson labs; 1∶5,000). Then the blots were stripped and incubated with anti-Rbt51 and secondary anti-rabbit-HRP antibody (Sigma; 1∶5,000)

### Recombinant protein expression and purification

The various rPga7 and rRbt5 derivatives were expressed as C-terminal Myc-6×His tagged proteins in the *P. pastoris* expression system as described (*Pichia* expression manual, Invitrogen, Carlsbad, CA). Briefly, cells from an overnight culture in BMGY were washed in 2 volumes of water and diluted in BMMY to OD_600_ 0.5–1. Cultures were then incubated at 28°C with vigorous shaking for 72 to 96 hours, with addition of 0.5% methanol every 24 hours. The cells were removed by centrifugation (1500 rpm, 30 min), 10 mM imidazole was added to the supernatant, and the pH was adjusted to 8.0. The medium was centrifuged again (13,000 rpm, 20 min), and the supernatant was filtered through a nitrocellulose 0.22 µm filter, then loaded onto a 2 ml HisTrap FF affinity column (GE Healthcare) equilibrated with 300 mM NaCl and 50 mM NaH_2_PO_4_, pH = 8. The column was washed every 150 ml load with 20 mM imidazole in 300 mM NaCl and 50 mM NaH_2_PO_4_, pH = 8, then the proteins were eluted with 250 mM imidazole in 300 mM NaCl and 50 mM NaH_2_PO_4_, pH = 8. To remove bound heme and obtain pure apo-protein, the proteins were treated with 3 M imidazole by tilting for 2 hours at 4°C, followed by centrifugation for 30 minutes at 4°C, until all heme was precipitated and the supernatant was clear. The supernatant was then filtered and loaded onto a HiLoad 16/60 Superdex size exclusion column (GE Healthcare) equilibrated with PBS.

### Hemin-bead association/dissociation assays

#### Association

Proteins (750 nM) in PBST (PBS +0.005% Tween 20) buffer were assessed for their ability to bind hemin-agarose vs. glutathione-agarose columns (Sigma). Beads and protein were mixed for 2 hours on a rotator at room temperature. Beads were extensively washed with PBST, resuspended in 2× Laemmli Sample Buffer +4% β-ME, and heated for 10 minutes at 100°C before loading onto precast 4–20% TGX gels (BioRad). Proteins were transferred by Western Blot to immobilon transfer membrane (Millipore), which was reacted with the anti-Myc 9E10 antibody (1∶1,000) followed by the anti-mouse IRDye 800 antibody (LI-COR; 1∶10,000), and detection by Odyssey Infrared Imaging System (LI-COR).

#### Dissociation

Recombinant proteins from *P. pastoris* growth medium were immobilized onto hemin-agarose for 2 hours as described above. The beads were then washed with PBS and resuspended in the indicated buffers (Glycine-HCl pH = 2.2, Citrate-Phosphate pH = 4.2, Citrate-Phosphate pH = 5.2, Citrate-Phosphate pH = 6.2, Tris-HCl pH = 7.2, Tris-HCl pH = 8.2, Carbonate-Bicarbonate pH = 9.2, Carbonate-Bicarbonate pH = 10.2, imidazole as indicated in PBS) and incubated for 1 hour. The supernatant was separated by centrifugation, and 5× Laemmli Sample Buffer was added. Beads were washed with PBS and resuspended in 2× Laemmli Sample Buffer in the same volume as the corresponding supernatant sample. Samples were gel-separated and detected as above.

### Heme extraction from hemoglobin

Bovine hemoglobin was covalently bound to CnBr-activated Sepharose-4B beads (Sigma). The hemoglobin (Hb) beads were washed extensively before incubation with Pga7 and Rbt5 to remove any spontaneously released heme. The experiment was done in triplicate for each sample. A UV-Vis spectrum of 50 µM apo-proteins was measured using Thermo Scientific NanoDrop2000 (BD bioscience), then 50 µl of the protein solution, or of buffer alone, was mixed with 10 µl Hb-beads. The samples were tilted for 30 minutes at room temperature, and UV-Vis spectrum of the supernatant was taken as above. The average spectrum of the triplicate sample was plotted.

### Isothermal Titration Calorimetry (ITC)

Titrations were performed at 25°C using a MicroCal iTC200 system (GE HealthCare). Proteins were in PBS and hemin was freshly prepared and diluted in PBS. All injections were carried out at 150 second intervals. To prevent/minimize heme adsorption, the calorimeter cell and the micro syringe used for injections were extensively washed with 10 N NaOH after each experiment. Protein concentration was determined using a NanoDrop spectrophotometer, and heme stock concentration was determined by absorbance at 398 nm in organic solution [61]. For the titration experiments, the concentrations of recombinant protein was 60 µM in the syringe and 20 µM hemin in the cell for rRbt5 titration and 10 µM hemin in the cell for rPga7 titration. Each titration was repeated at least 3 times with different protein batches.

The resulting titration data were analyzed and fitted using the Origin for ITC software package supplied by Microcal to obtain the stoichiometry (n), the dissociation constants (*K_D_*), the enthalpy (ΔH) and the entropy (ΔS) changes of binding. For the fit, any constraints on the stoichiometry and ΔH were not fixed.

### Absorption spectroscopy

UV–visible absorption spectra of hemin titrated into PBS buffer or into a Pga7 solution was collected using a Cary 50 scanning spectrophotometer (Varian), and a 1-cm-pathlength 3 ml quartz cuvette. Heme stock concentration was measured as described above. The Pga7 concentration was determined by the absorbance of Pga7 at 280 nm using predicted absorption coefficients of ε = 26,600 mM^−1^. In order to calculate the stoichiometry, the relative absorbance at 406 nm of Pga7-heme minus heme alone was plotted against the heme concentration.

### Size exclusion chromatography

100 µl samples were injected in a Superdex 200 10/300 column (GE Healthcare) equilibrated with PBS, and monitored on an AKTA Avant system. Absorbance was recorded at 280 nm and 406 nm.

### Nuclear Magnetic Resonance

NMR experiments were performed on protein samples ranging from 0.1 to 0.5 mM concentration in PBS buffer, pH 7.4. Monodimensional ^1^H NMR spectra tailored to detection of paramagnetic signals were acquired at 600 MHz (14.1 T) with a spectral width of 200 ppm and 64 K data points. Water suppression was achieved by fast repetition rate and presaturation during a 100-ms recycle delay.

### SPR measurements

All measurements were performed using a Biacore T200 apparatus (GE). Proteins were subjected to size exclusion chromatography to remove any trace of aggregation. The CFEM proteins were immobilized directly onto a CM-5 chip using standard amine chemistry. All injections were performed multiple times in random order and double-blanked against an empty cell and against buffer alone. Data analysis was performed using Biacore's standard evaluation software.

## Supporting Information

Figure S1 **Alignment of the signal peptide-CFEM domain region of the five most closely related** ***C. albicans*** **CFEM proteins, Rbt5, Rbt51/Pga10, Csa2, Csa1 (1^st^ CFEM domain only) and Pga7.** The sequences were aligned using the MAFFT G-INS-i algorithm and visualized with Jalview [64]. The location of the FLAG epitope introduced into Rbt5 and Pga7 is indicated with a green arrowhead. The conserved aspartic acid mutagenized in Rbt5 and Pga7 is indicated with a red arrowhead. The predicted GPI anchor site of Rbt5 and Pga7 is indicated with an orange arrowhead.
(TIF)

Figure S2 **The** ***ccc2^−/−^ pga7^−/−^*** **strain is strongly defective in heme and hemoglobin-iron utilization.** The indicated *C. albicans* CFEM protein deletion strains in *ccc2-/-* background were grown in iron-limiting conditions (YPD +1 mM ferrozine) in the presence of increasing concentration of either bovine hemoglobin (A) or hemin (B) as a sole source of iron. Optical density was measured after 3 days at 30°C. Error bars represent standard deviations of triplicates. The strains used were KC68 (*ccc2^−/−^*), KC139 (*ccc2^−/−^ rbt5^−/−^*), KC170 (*ccc2^−/−^ rbt5^−/−^ rbt51^−/−^ csa1^−/−^*), KC485 (*ccc2^−/−^ pga7^−/−^*), KC508 (*ccc2^−/−^ rbt5^−/−^ rbt51^−/−^ csa1^−/−^ pga7^−/−^*).
(TIF)

Figure S3 **FLAG-tagged Pga7 and Rbt5 are fully active** ***in vivo*** **.** The ability of the FLAG-tagged alleles of *RBT5* and *PGA7* to complement their respective deletions was compared to complementation with the native alleles of these genes, by growing the strains in iron-limiting conditions (YPD +1 mM ferrozine) in the presence of increasing concentration of bovine hemoglobin as a sole source of iron. Optical density was measured after 3 days at 30°C. Error bars represent standard deviations of triplicates.
(TIF)

Figure S4 **Stability of Pga7 and Rbt5 binding to hemin-agarose.** The recombinant proteins Rbt5_23–219_ and Pga7_18–195_ were immobilized on hemin-agarose beads. Release of Rbt5_23–219_ and Pga7_18–195_ from hemin-agarose was tested at different pH (A) and different imidazole concentrations (B). B = bound fraction, S = supernatant (released) fraction.
(TIF)

Figure S5 **Stable heme binding by Rbt5 and Pga7.** apo-Rbt5_23–219_ or apo-Pga7_18–195_ (50 µM) were subjected to size exclusion chromatography before (top panels) or after (bottom panels) a 5 min incubation with 25 µM heme. Absorbance was measured at 280 nm (blue curves) and 406 nm (red curves), representing protein and heme absorbance, respectively.
(TIF)

Figure S6 **Apo-Pga7 interacts with apo-Rbt5 but not with apo-Pga7.** SPR analysis was carried out by immobilizing the recombinant CFEM proteins Pga7_18–195_ (green), Rbt5_23–219_ (blue), and the D72A mutant of Rbt5_23–219_ (red) on a biosensor chip. 1.25 µM apo-Pga7_18–195_ was injected for 120 sec over all three surfaces.
(TIF)

Figure S7 **Proximity tree of Pga7 and Rbt5 homologues in six fungal genomes.** The genomes of five species were screened for sequences similar to *C. albicans* Rbt5, Rbt51 and Pga7 by BLAST. The 18 most homologous sequences were aligned using the MAFFT G-INS-i algorithm [64] and a tree was built on this alignment using the NJ method. Prefixes: no prefix – *C. albicans*; Cp – *C. parapsilosis*; Ct – *C. tropicalis*; Clus – *C. lusitaniae*; Dh – *Debaryomyces hansenii*; Lelon – *Lodderomyces elongisporus*.
(TIF)
