# Supplementary figures and images for: A Relay Network of Extracellular Heme-Binding Proteins Drives C. albicans Iron Acquisition from Hemoglobin
Source: PLoS Pathog. 2014 Oct 2;10(10):e1004407. doi: 10.1371/journal.ppat.1004407 (PMC4183699; doi:10.1371/journal.ppat.1004407)

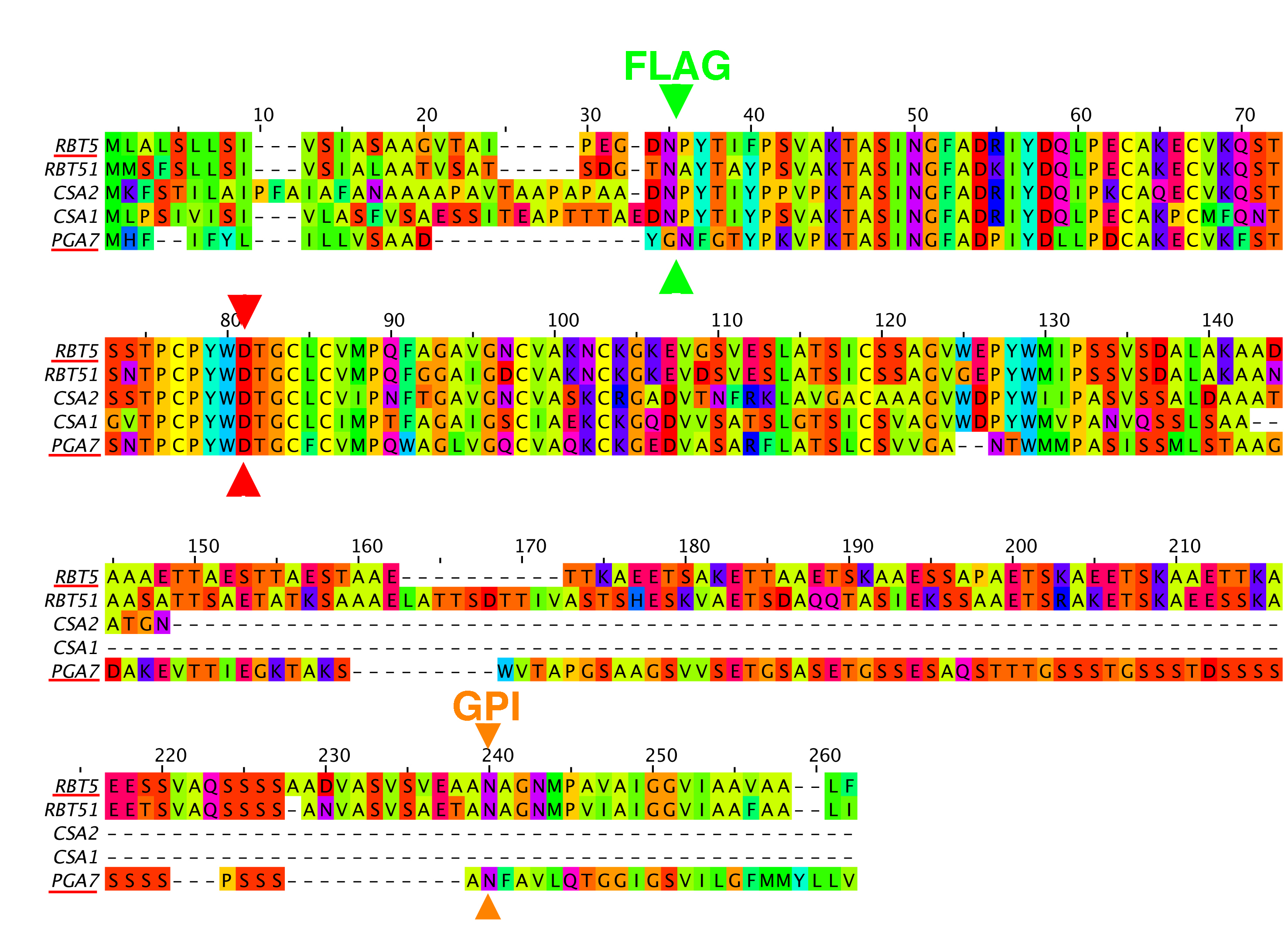

Supplement: Figure S1 — Alignment of the signal peptide-CFEM domain region of the five most closely related C. albicans CFEM proteins, Rbt5, Rbt51/Pga10, Csa2, Csa1 (1st CFEM domain only) and Pga7. The sequences were aligned using the MAFFT G-INS-i algorithm and visualized with Jalview [64]. The location of the FLAG epitope introduced into Rbt5 and Pga7 is indicated with a green arrowhead. The conserved aspartic acid mutagenized in Rbt5 and Pga7 is indicated with a red arrowhead. The predicted GPI anchor site of Rbt5 and Pga7 is indicated with an orange arrowhead. (TIF) [file ppat.1004407.s001.tif]

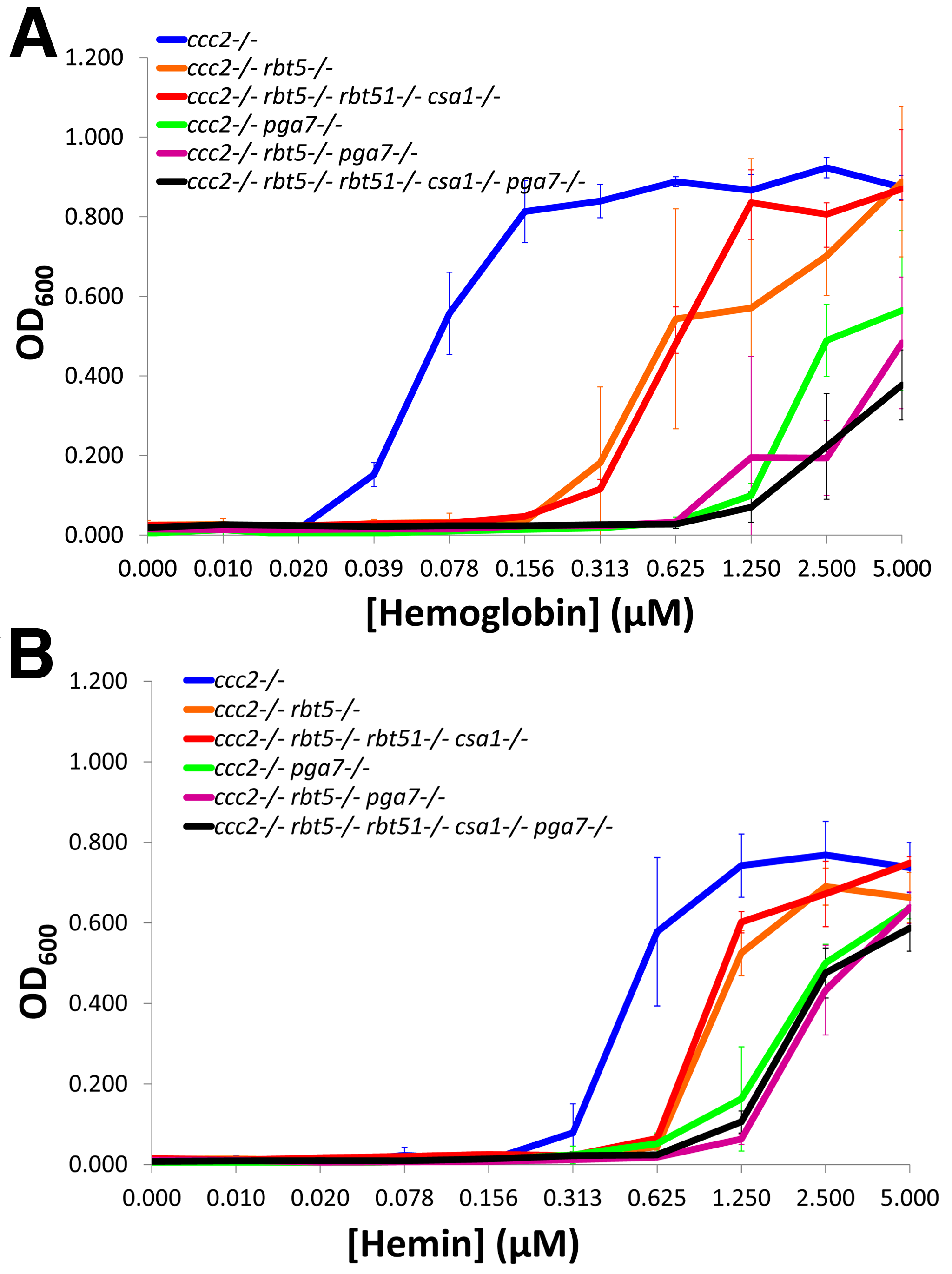

Supplement: Figure S2 — The ccc2−/− pga7−/− strain is strongly defective in heme and hemoglobin-iron utilization. The indicated C. albicans CFEM protein deletion strains in ccc2-/- background were grown in iron-limiting conditions (YPD +1 mM ferrozine) in the presence of increasing concentration of either bovine hemoglobin (A) or hemin (B) as a sole source of iron. Optical density was measured after 3 days at 30°C. Error bars represent standard deviations of triplicates. The strains used were KC68 (ccc2−/−), KC139 (ccc2−/− rbt5−/−), KC170 (ccc2−/− rbt5−/− rbt51−/− csa1−/−), KC485 (ccc2−/− pga7−/−), KC508 (ccc2−/− rbt5−/− rbt51−/− csa1−/− pga7−/−). (TIF) [file ppat.1004407.s002.tif]

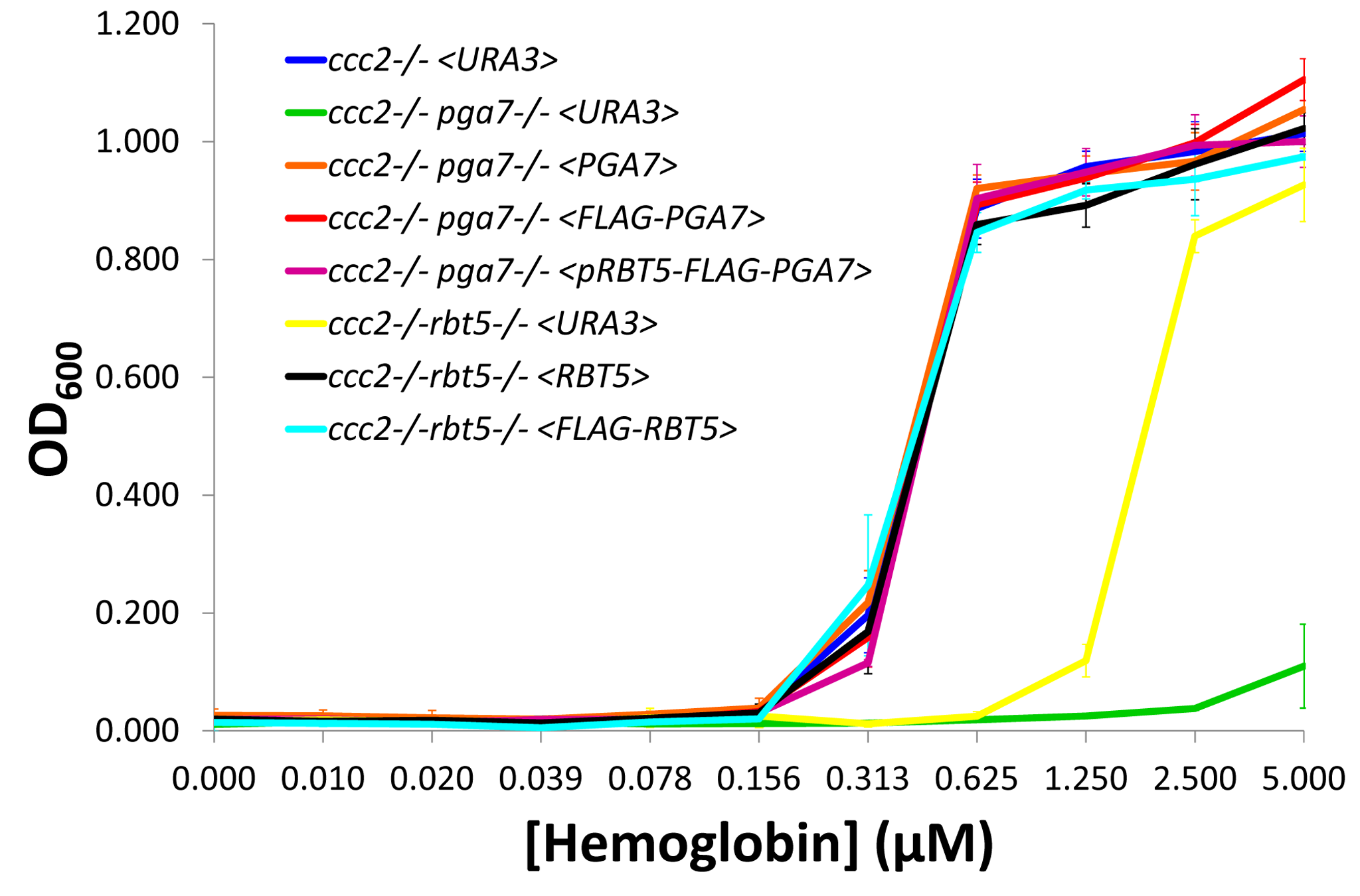

Supplement: Figure S3 — FLAG-tagged Pga7 and Rbt5 are fully active in vivo . The ability of the FLAG-tagged alleles of RBT5 and PGA7 to complement their respective deletions was compared to complementation with the native alleles of these genes, by growing the strains in iron-limiting conditions (YPD +1 mM ferrozine) in the presence of increasing concentration of bovine hemoglobin as a sole source of iron. Optical density was measured after 3 days at 30°C. Error bars represent standard deviations of triplicates. (TIF) [file ppat.1004407.s003.tif]

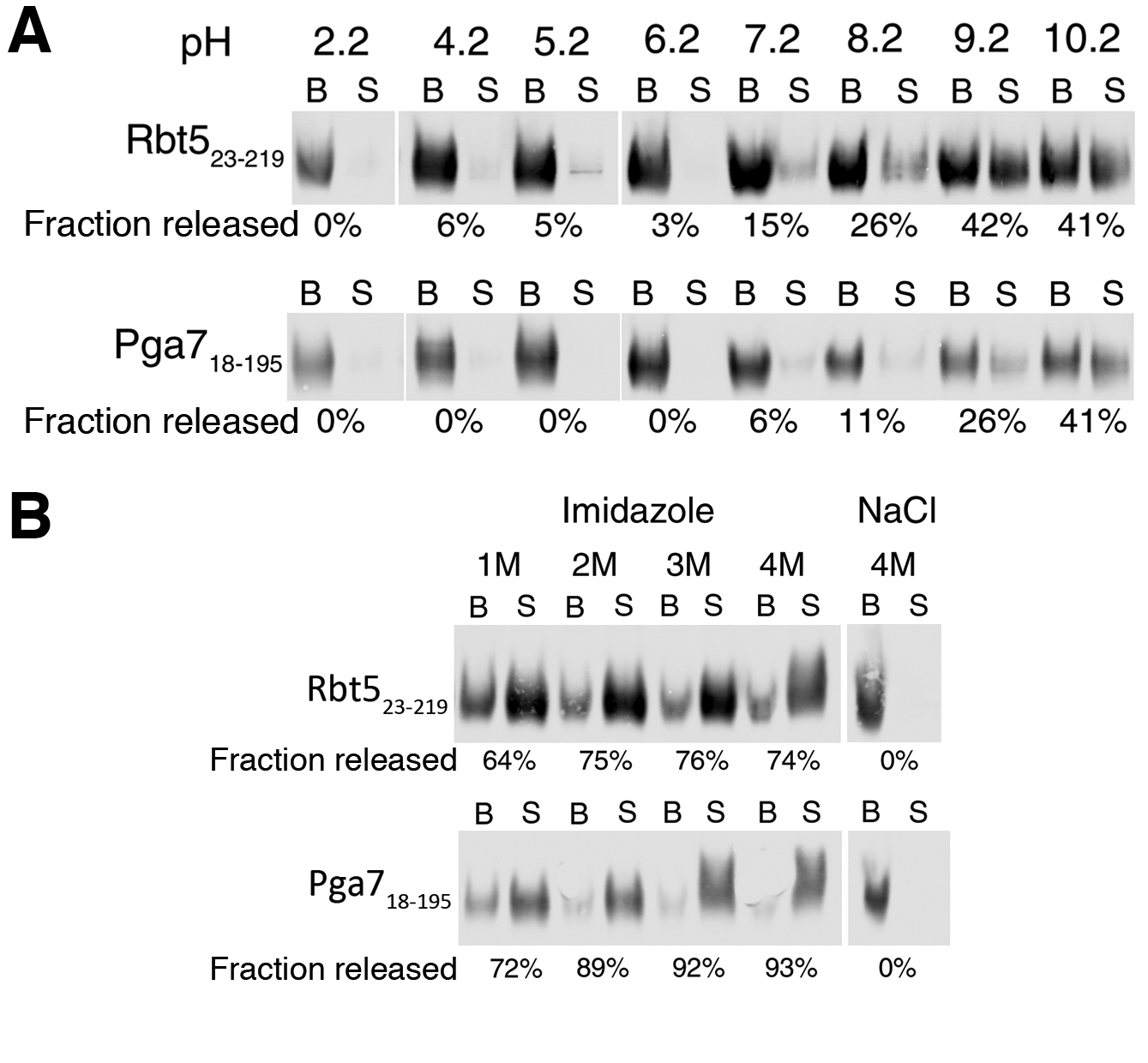

Supplement: Figure S4 — Stability of Pga7 and Rbt5 binding to hemin-agarose. The recombinant proteins Rbt523–219 and Pga718–195 were immobilized on hemin-agarose beads. Release of Rbt523–219 and Pga718–195 from hemin-agarose was tested at different pH (A) and different imidazole concentrations (B). B = bound fraction, S = supernatant (released) fraction. (TIF) [file ppat.1004407.s004.tif]

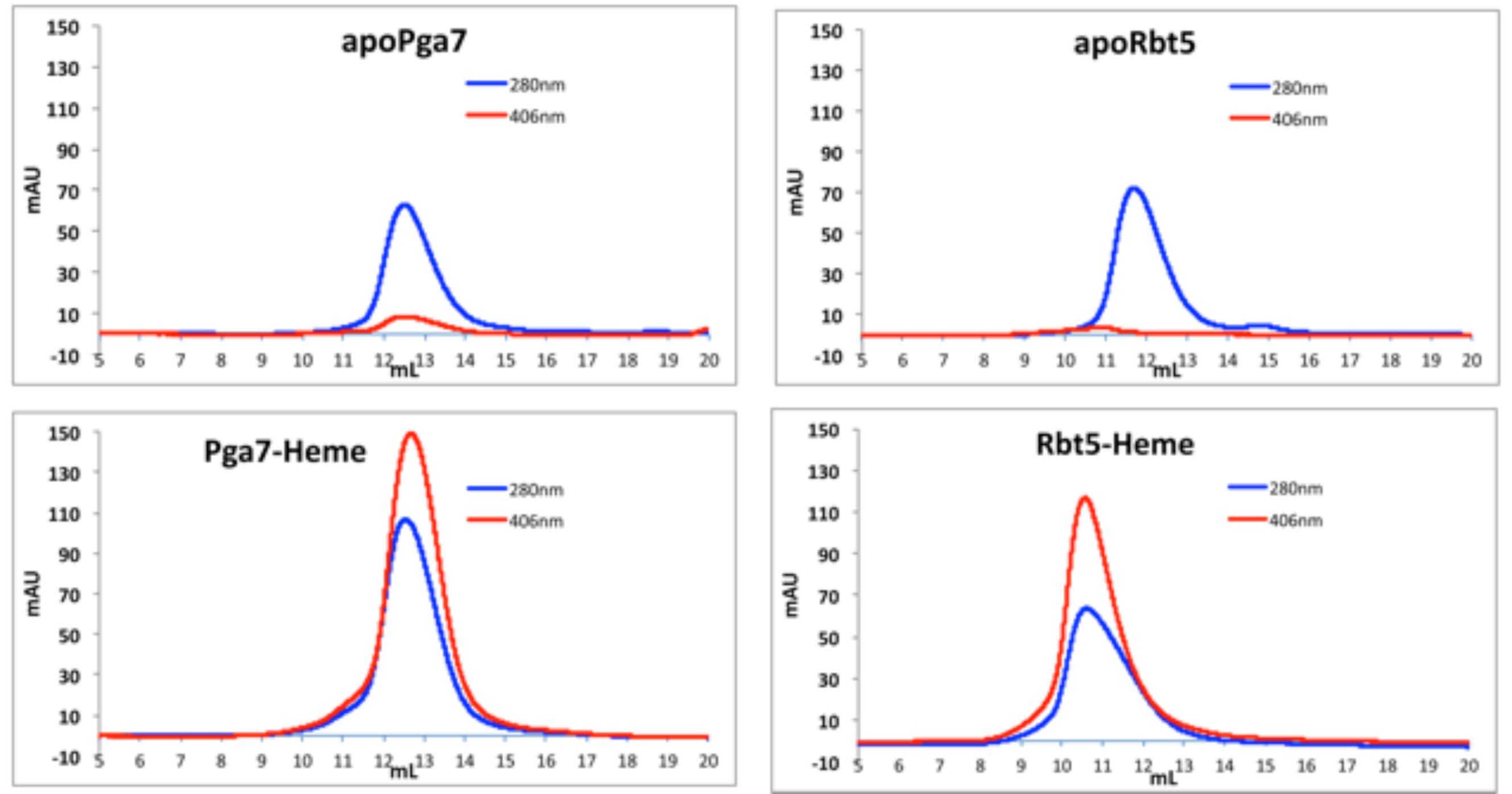

Supplement: Figure S5 — Stable heme binding by Rbt5 and Pga7. apo-Rbt523–219 or apo-Pga718–195 (50 µM) were subjected to size exclusion chromatography before (top panels) or after (bottom panels) a 5 min incubation with 25 µM heme. Absorbance was measured at 280 nm (blue curves) and 406 nm (red curves), representing protein and heme absorbance, respectively. (TIF) [file ppat.1004407.s005.tif]

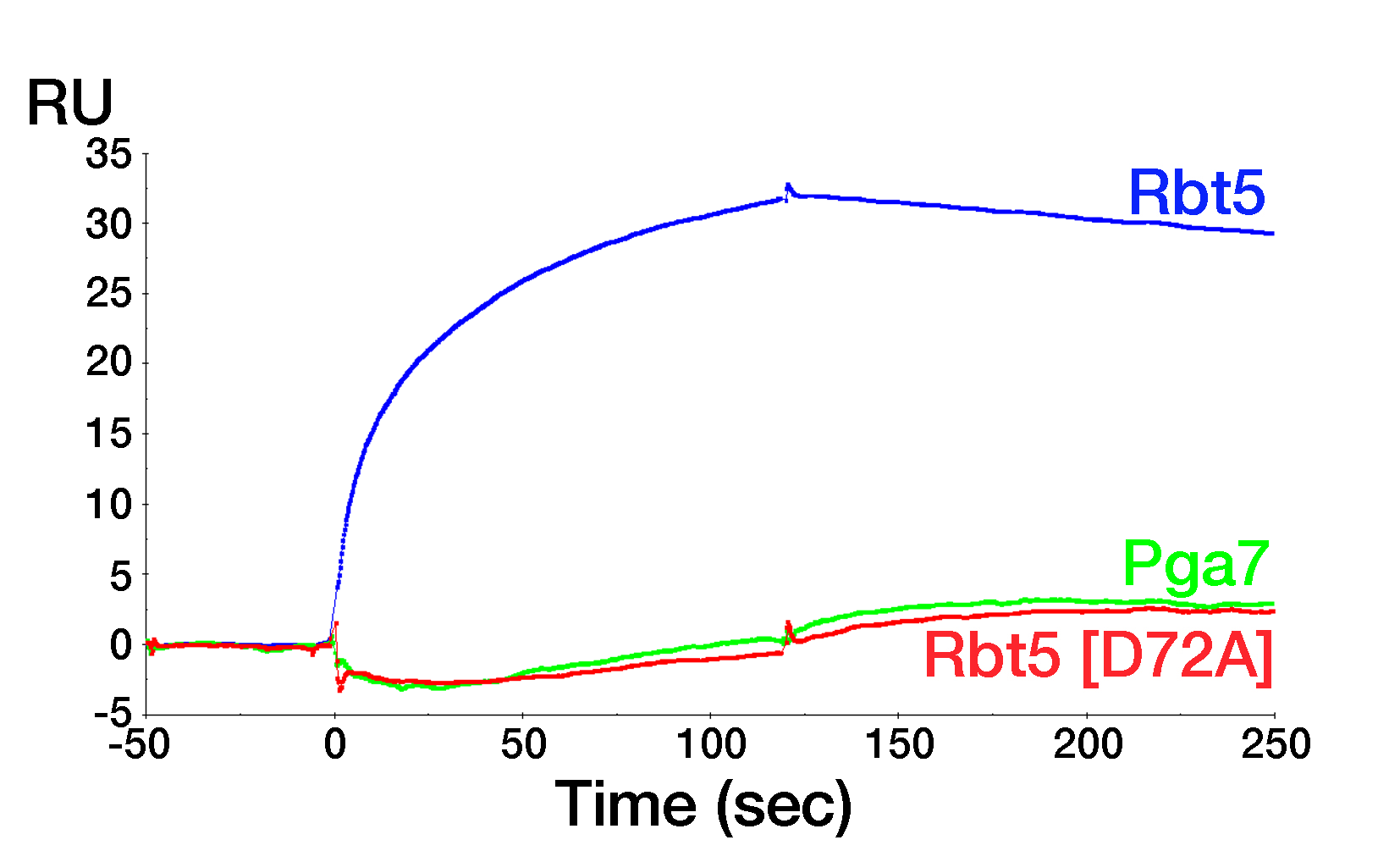

Supplement: Figure S6 — Apo-Pga7 interacts with apo-Rbt5 but not with apo-Pga7. SPR analysis was carried out by immobilizing the recombinant CFEM proteins Pga718–195 (green), Rbt523–219 (blue), and the D72A mutant of Rbt523–219 (red) on a biosensor chip. 1.25 µM apo-Pga718–195 was injected for 120 sec over all three surfaces. (TIF) [file ppat.1004407.s006.tif]

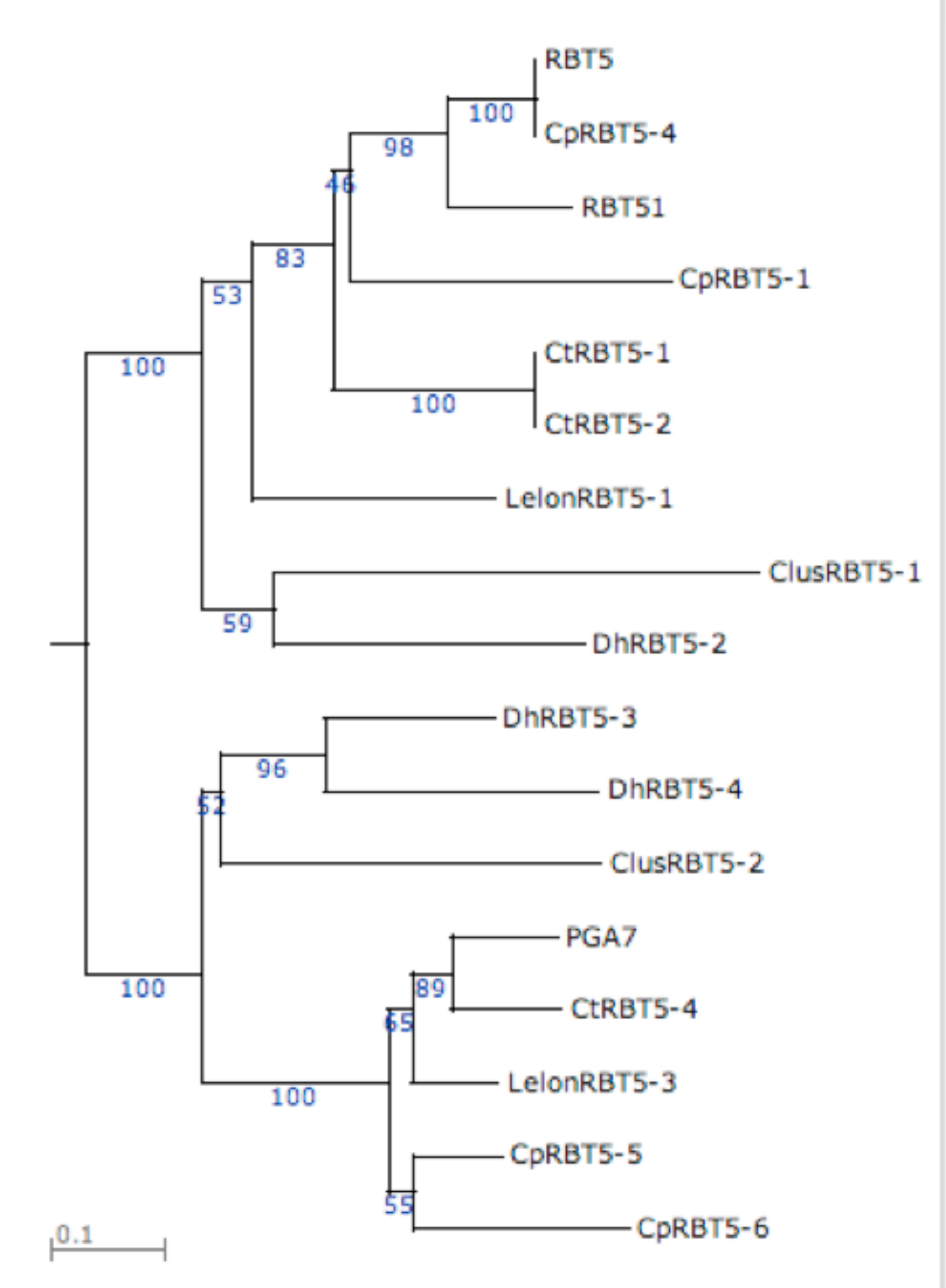

Supplement: Figure S7 — Proximity tree of Pga7 and Rbt5 homologues in six fungal genomes. The genomes of five species were screened for sequences similar to C. albicans Rbt5, Rbt51 and Pga7 by BLAST. The 18 most homologous sequences were aligned using the MAFFT G-INS-i algorithm [64] and a tree was built on this alignment using the NJ method. Prefixes: no prefix – C. albicans; Cp – C. parapsilosis; Ct – C. tropicalis; Clus – C. lusitaniae; Dh – Debaryomyces hansenii; Lelon – Lodderomyces elongisporus. (TIF) [file ppat.1004407.s007.tif]
